# Arts engagement trends in the United Kingdom and their mental and social wellbeing implications: *HEartS Survey*

**DOI:** 10.1371/journal.pone.0246078

**Published:** 2021-03-12

**Authors:** Urszula Tymoszuk, Neta Spiro, Rosie Perkins, Adele Mason-Bertrand, Kate Gee, Aaron Williamon

**Affiliations:** 1 Centre for Performance Science, Royal College of Music, London, United Kingdom; 2 Faculty of Medicine, Imperial College London, London, United Kingdom; University of Hong Kong, HONG KONG

## Abstract

Evidence on the role of the arts in promoting health and wellbeing has grown over the last two decades. In the United Kingdom, studies using secondary data sources have documented temporal variations in levels of arts engagement in the population, its determinants and its mental wellbeing implications. However, arts engagement is often characterized by prioritizing “high-brow” art forms. In this article, we introduce the *HEartS Survey*, a tool that aims to increase the balance between inclusivity and brevity of existing arts engagement measures and to focus specifically on the connection between arts engagement and social wellbeing. We explore trends in participatory and receptive engagement with literary, visual, performing, crafts and decorative arts among 5,338 adults in the UK in 2018–2019 using summative engagement scores and cluster analysis. Regression models, adjusted for demographic, socioeconomic, health, and social covariates, examine correlations between arts engagement and psychological and social wellbeing measures. Over 97% of respondents reported engagement in one or more arts activities at least once during 2018–2019, with reading and listening to music being the most popular activities. Arts engagement grouped into three distinct clusters: 19.8% constituted “*low engagers*” whose main source of engagement was occasional reading; 44.4% constituted “*receptive consumers*” who read and listened to music frequently and engaged with popular receptive arts activities such as cinema, live music, theater, exhibitions, and museums; and 35.8% constituted “*omnivores*” who frequently engaged in almost all arts activities. In agreement with existing studies, more arts engagement was associated with higher levels of wellbeing, social connectedness, and lower odds of intense social loneliness. In contrast, we found a positive association between more arts engagement, depression, and intense emotional loneliness for the most highly engaged *omnivores*. We conclude that arts engagement in the population forms specific profiles with distinct characteristics and consider implications for mental and social wellbeing.

## Introduction

Worldwide, engagement with the arts is routinely monitored by governments and cultural institutions in order to understand participation trends and impacts on health and wellbeing. In the United Kingdom (UK), the past two decades have seen routine national surveys of the adult population’s engagement with the arts, culture, and sport, including the *Taking Part* survey [1], and their connections with wellbeing [2]. In 2008, the epidemiological panel cohort study *Understanding Society*, based on 40,000 UK households, was launched, and questions on adults’ engagement with arts and culture adapted from *Taking Part* were included in Wave 2 (2010–2012) and Wave 5 (2013–2015). In England, a limited number of questions on arts engagement have been asked in other ongoing cohort studies of specific populations such as *The Whitehall II Study*, a cohort of 10,308 British Civil Service employees recruited in 1985, and *The English Longitudinal Study of Ageing (ELSA)*, a cohort of non-institutionalized English people aged 50 years and older at enrolment in 2002–2003. These large scale studies incorporated measures of some aspects of wellbeing, with a focus on depression and mental health [e.g. 2–5] and some more limited aspects of loneliness and social connectedness.

Data from these large-scale, ongoing, and often nationally representative cohort studies can be transformative for understanding arts engagement and the relationship with wellbeing in the population. However, differences in definitions of arts and culture and inconsistent analytical approaches can result in inconsistent representations. These differences can lead to confusion concerning patterns of arts engagement and the relationship between arts engagement and mental and social wellbeing. This article therefore re-examines the possible representations of arts engagement, compares two different analytical approaches on the same data, and investigates the relationship between arts engagement and measures of mental and, in particular, social wellbeing.

### Representations of arts engagement

A small number of reports illustrate the inconsistencies in arts engagement representation. For example, a report based on data from *Understanding Society* found that, in 2013 and 2014, 76% of adults in England participated in at least one arts activity and 61% attended at least one arts event [6]. During the same time period, a report based on data from *Taking Part* found that 77.5% of adults engaged with at least one arts activity in the last year, with 37.7% engaging with both arts events and participatory arts, 30.7% only attending arts events, and 9.2% only participating in arts activities [7]. Another study based on 2010–2012 data from *Understanding Society* described 89.7% of the UK population as “disengaged” with participatory performing, visual, and literary arts and 47.7% as “rarely engaged” with arts events and cultural places [8]. Finally, a study looking across three waves of *Taking Part* (2005–2006, 2008–2009, 2010–2011), and using a broad definition of engagement, reported that only 8.7% of the English population engage with state-supported forms of culture and arts, while only 11% are disengaged from most mainstream leisure and culture [9].

It is possible that such inconsistencies result from conceptual differences and overlaps between *arts*, *cultural*, and *leisure* activities. Previous lists of arts and cultural activities have been affected by a number of factors (see also [10–12]). These include the definitions of arts and culture used, the priorities of the research project or cohort study, as well as practical limits such as space and participant burden in cases of online or telephone survey studies. Nonetheless, the current approaches to conceptualization and measurement of arts engagement in the UK have been shaped by *Taking Part*’s inquiry into predominantly state-sponsored arts activities [9]. Despite differences in the length or diversity of arts activity in existing lists, certain trends can be identified. These trends can be seen in conceptualization and quantification of arts engagement as well as inconsistencies regarding the nature of engagement, the scope of art production and engagement, and the social context of engagement. These observations were key in developing our new approach to representing and analyzing arts engagement.

It is common to separate the nature of such activities between *receptive engagement*, art that has been created and is now experienced by a listener, audience member, or gallery visitor, and *participatory engagement*, the creation of and participation in the arts. A bias toward “highbrow” and “formal” interpretation of arts made or performed by highly skilled creators and state-sponsored art forms is often revealed in this approach with a greater representation of receptive compared with participatory engagement in some studies [9, 13]. For example, although *ELSA* includes questions about reading a daily newspaper, types of programmes watched on television, and having a hobby or pastime, arts and cultural engagement tends to be analysed using three items: the frequency of visits to (1) the cinema, (2) the art gallery or museum, and (3) the theater, a concert, or the opera [3, 14]. This approach can lead to less attention being paid to amateur, every day creation and participation, which tends to be more accessible and, thus, may lead to an understimation of arts engagement in the population. For instance, a report based on *Understanding Society* data ascribed its higher rates of participatory arts engagement between 2013 and 2014 compared with those found for the same time period in the *Taking Part* survey, to the inclusion of a “reading for pleasure” item [6].

There is also a difference in the number of arts activities included in arts engagement in surveys. 21 participatory arts activities and 19 receptive activities were included in one report [6] based on *Understanding Society* data, while 16 participatory arts activities and 13 receptive activities were in another report using *Taking Part* data [7]. Despite these lengthy lists, the surveys did not include a question on listening to recorded music on personal devices and digital streaming platforms, despite their popularity [15]. Indeed, arts engagement assessment in ongoing national surveys appears particularly out of sync with trends in digital, on-demand video and audio content consumption such as music streaming services, podcasts, and audiobooks [15, 16].

Further inconsistencies relate to explicit mentions of the scope or modes of art production and specific art genres in the definitions of arts engagement used in surveys. For example, in *Taking Part* and *Understanding Society*, “reading for pleasure” excludes reading “newspapers, magazines or comics”, and visiting arts and crafts exhibitions exclude “crafts markets”. The item covering singing prioritizes public performance, and perhaps formal public performance, by excluding karaoke (e.g. “sang to an audience or rehearsed for a performance (not karaoke)”). However, much artistic engagement–and not least singing–can occur in a variety of settings in which “rehearsal” or “performance” are not relevant (e.g. singing at home with friends or family and in therapeutic settings). Similarly, certain genres are explicitly mentioned in definitions, for instance *Taking Part* and *Understanding Society* provide a single example of dance genre, ballet; two items on attending musical performances specify “classical music” and “rock, pop or jazz”; and three items on attending dance performances specify “ballet”, “contemporary dance”, and “African people’s dance or South Asian and Chinese dance”. Furthermore, modes of art production are inconsistently explicit across the art forms and activities (e.g. “Used a computer to create original artworks or animation” or “Played a musical instrument” vs. “Written any stories, plays or poetry”). These explicit mentions, although perhaps making the questions more specific, draw attention to examples and potentially bias respondents’ recall. Overall, most mentions tend to reinforce a bias towards “highbrow”, “formal”, or “state-sponsored” arts activities and may contribute to underrepresentation of arts engagement in the population [17].

Another inconsistency relates to the extent to which the social context of arts engagement is explicitly questioned. For instance, items such as “been a member of a book club, where people meet up to discuss and share books” or “taken part in a carnival or street arts event (e.g. as a musician, dancer or costume maker)” in *Understanding Society* or a question on involvement in “social indoor games, cards, bingo, chess” in *The Whitehall II* study explicitly include shared, social elements and the setting in the description of the activity. Overall, there is also an assumption that certain arts activities are intrinsically more social than solitary or individual in their nature and purpose of engagement, for instance attending art events versus reading or collecting hobbies [18, 19]. However, to our knowledge there are no large scale survey data on how, in basic social terms, the respondents consider themselves as engaging with artistic and cultural activities: mainly alone, mainly socially, or somewhere in the middle.

Conceptualization of arts engagement is shaped further by how it is operationalized analytically. Most national surveys and studies stemming from UK-based epidemiological cohorts ask about the frequency of engagement for each listed art activity in the last 12 months, and the frequency usually ranges between “at least weekly” to “less than once a year”. One common approach to quantifying arts engagement is to create a composite score of arts activities with an ordinal scale of engagement frequency [3, 6, 7]. Another approach is to derive profiles–or clusters–of arts engagement in the population [8, 9]. In both approaches respondents who do not engage, or engage less than others are compared with those who engage more, often in relation to their socioeconomic characteristics or health and social outcomes [8, 14, 20]. These different ways of operationalizing arts engagement are often implicit in analysis, and there are few attempts to scrutinize the implications of these decisions.

Noting the trends in arts engagement survey design, there remains a need for a greater balance between inclusivity and brevity in how arts engagement is measured in large-scale surveys. This article introduces the *HEartS Survey* (Health, Economic, and Social impacts of the ARTs), which is designed to chart current arts engagement in the UK and to explore its sociodemographic characteristics and correlations with mental and social wellbeing. It is based on existing tools and adapted to remove emphases on modes of production, genres, and, where possible, settings of arts engagement.

### Relationships between arts engagement and social wellbeing

Having charted arts engagement, it is possible to explore correlations with outcomes on mental and social wellbeing. Evidence on the role of arts and cultural engagement in promoting health and wellbeing is ever growing [12, 21]. The relationship between arts engagement and mental health–in particular, wellbeing and depression–has been well-rehearsed in large-scale epidemiological data [2–4, 20], studies on individual arts activities [22–24], and literature reviews [e.g., 25–29]. Despite the suggested importance of the connection between arts engagement and social outcomes specifically, and indications that the arts may play a role in preventing or alleviating loneliness and boosting social connectedness and social wellbeing [30–33], the arts have been less represented in large-scale epidemiological cohorts (compared with, for example, mental and physical health outcomes) and have been less frequently documented in empirical research. This is a particularly important area given that, in England alone, 5% of adults have reported feeling lonely “often/always”, a further 16% “sometimes”, and 24% “occasionally”, making this painful emotional experience a reality for almost half of the population in 2016 and 2017 [34]. In this study, we therefore pay particular attention to the impact of arts engagement on social outcomes, including both loneliness and social connection, and place these in context of mental wellbeing and depression outcomes.

Feelings of loneliness occur when our intimate and social needs are not met well enough [35]. Feeling lonely is not the same as being alone but instead involves feeling isolated, disconnected, and not belonging. These feelings are seen as reflecting the mismatch between desired and actual relationships [36]. Social connectedness is about how we think of our relationships to other people including our relationships with other individuals and wider groups. Rather than looking at the deficit notion of loneliness, social connectedness can be seen as “a subjective evaluation of the extent to which one has meaningful, close, and constructive relationships with others (i.e., individuals, groups, and society)” [37, p. 43].

Studies focussing on specific arts and cultural activities and interventions associated with specific diagnoses or concerns have suggested the place of the arts in reducing feelings of loneliness and/or enhancing social connections. In music, a randomised controlled study suggested an effect of music therapy on loneliness among institutionalized older adults with mild depression compared with controls [38]. Music interventions involving drumming have, in qualitative studies, been reported to enhance feelings of togetherness, belonging, and connectedness between mental health service users and their carers [23] and among soldiers experiencing PTSD [39]. In museums, qualitative research on art and reminiscence workshops with mentally vulnerable adults and lonely older adults suggested that they can support social inclusion and community building [40], and museum programmes can help to reduce isolation and loneliness for socially isolated older adults through creating opportunities for social inclusion, social engagement, and enhanced communication [41]. Art therapy in art museums has been seen, in another qualitative study, to promote social connectedness in older adults [42]. A multi-method observational study has suggested that community arts programmes, including crafts activities such as painting, pottery, and printmaking, are effective in addressing loneliness and contributing to feelings of social inclusion among socially isolated older adults [43].

In examples from non-diagnosis-led activities, several studies focussing on particular activities suggest their positive effects on loneliness and social connectedness. For example, self-report questionnaire data suggested that participants in adult education singing and creative writing classes experienced a more rapid increase over time in relational bonding (as seen in the social network density and the proportion of their classmates that they could name, felt connected with, and talked to during class) compared with those in craft classes [44, 45]. One hour of choral singing by amateur choristers was also shown to improve social connectedness scores compared with not singing and compared with only half an hour of singing [46]. In a study that used both a loneliness scale and measurement of engagement in social activities, community choral singing was found to reduce loneliness in older adults [47]. Dance, in an interview based study of line dancing by women aged over 60 years, was suggested to increase social activity [48]. A mixed method study on textile crafting has also been linked with social interaction and connectedness [49], and a qualitative study has suggested connection between textile crafting and social connectedness through belonging [50].

Many of these studies on loneliness and social connectedness focus on older adults. This focus is even more apparent in review articles and large-scale epidemiological studies. Indeed, reviews have indicated that participatory arts can strengthen and build relationships [51] and promote reciprocal relationships among older adults, care givers, and the wider community [52]. Community-engaged art programs have been seen as promising for addressing loneliness in older adults [53]. Large scale epidemiological studies have suggested that frequent engagement with museums, galleries, and exhibitions may be a protective factor against loneliness in older adults [14]. More broadly, one study suggested that some arts activities (including reading books) but not others (e.g. watching TV, listening to the radio, and spending time on the computer (passive activities)) were linked to social connectedness in older people [54].

The importance of the social element of art engagement has been posited as relevant for several social outcomes. The concepts of social capital such as trust, norms, and networks that can foster social relationships and cohesion through “bonding” and “bridging” [55–57] have been suggested as particularly important in understanding the key processes shaping wellbeing and loneliness. One review [58] highlighted the particular importance of the notion of “bonding” [55, 56] (in the form of emotional support, belonging, and shared identity) in how participatory arts can help to connect people who share some characteristics (including common identity, situation). The same review pointed out that difficulties seen elsewhere, such as problems of inequality, exclusivity, are equally relevant and do not disappear in arts activities.

From another perspective, social engagement in the arts has been explicitly connected to improved prosocial behavior. For example, participatory music making in synchrony with others has been associated with more cooperative behavior [59]. Synchrony, entrainment, and mimicry have been posited as key among the mechanisms at play in the relationship between music and prosociality as well as shared goals and intentions, and self-other merging and the release of endorphins [60, 61].

Nevertheless, some lone, receptive, activities have also been seen to reduce loneliness. For example, music listening was an activity chosen with the goal of reducing loneliness in an online survey in university students [62] and in an interview study with older adults [63]. Similarly, in an interview study, reading for pleasure has been associated with reduced loneliness in older adults [64]. Mechanisms underlying these effects have been posited to be shared with those relevant to in-person interaction with others or a replacement for being with others. For example, music listening has been suggested to act as a social surrogate, with listeners resorting to “temporary substitutes, so-called social surrogates, if direct social interaction is not possible” [65, p. 232]. Another theory about the potential role that receptive activities, in this case reading, can play in coping with loss, and therefore coping with loneliness, starts from the premise that it is possible to cope with loss directly or indirectly [64]. The direct approach relies on such external factors (such as social activities and interactions) but the indirect approach is more appropriate in some cases. Indeed, the theory suggests that effective coping can be improved when individuals are free enough of external social factors that they do not depend exclusively on them for a sense of wellbeing. In this case those who engage in reading for pleasure may feel less lonely than those who do not [64].

Taken together these studies provide promising evidence for the role of a range of arts activities in loneliness and social connectedness. However, they illustrate that emphasis in research–especially in reviews and epidemiological studies–has been on older adults, with some focussing on loneliness *or* social connectedness. Several of the arts activities have been participatory, and it seems intuitive that participatory engagement would be particularly important for reducing loneliness and supporting social connectedness. However, a small number of studies point to the possibility that lone engagement in the arts may be equally important in at least some cases. Similar to research on arts engagement in general, many studies look at specific interventions or more formal activities in daily life, potentially excluding more informal, private activities.

In sum, the *HEartS Survey* addresses three overarching gaps in current knowledge: (1) it provides data on broadly-defined and inclusive arts engagement levels among adults, including younger and middle-aged adults, in the UK, (2) it enables comparisons of two different ways of operationalizing arts engagement in analyses, and (3) it investigates arts engagement in relation to mental *and* social wellbeing, including both positive and negative aspects (e.g. social connectedness and loneliness).

## Methods

### Study sample

UK-based respondents were recruited through an online data collection platform, Qualtrics, over a period of six months between March and August 2019. Data collection quotas were set for gender, age, geographical region, ethnicity, and education following the overall distributions of these key sociodemographic variables in the UK 2011 Census. A total of 11,861 respondents started the survey. Of these, 1,623 did not consent to participate in the survey and stopped at the consenting process. A further 3,219 respondents were excluded after answering initial sociodemographic questions as the quotas for their characteristics were already reached. A further 969 were excluded due to completing the survey in under four minutes (i.e. speeding through the survey, n = 97) or providing nonsense or abusive responses to open questions (n = 872). The remaining 6,050 participants formed the final study sample, all of whom were paid a modest fee for participating via the Qualtrics platform. Participants who responded “prefer not to say” to gender, education, living status, household income, health conditions, or self-reported health, and hence had missing data, were deleted from the analytical sample, yielding a final sample size of 5,338.

### Materials and procedure

Ethical approval for the research was granted by the Conservatoires UK Research Ethics Committee on 16 February 2019. Participants gave written informed consent to participate and links to support (e.g. for mental health) were provided at the end of the survey. The *HEartS Survey* consists of seven sections: (1) demographics, (2) arts and cultural activities, (3) open question on arts engagement and social connectedness, (4) mental health and wellbeing, (5) physical activity, (6) social wellbeing, and (7) household income and arts spending. The questions and scales included in the survey are provided in the (S1 File). During development, the survey was piloted within the Centre for Performance Science (n = 25) to elicit feedback on the suitability of the measures and the average completion time. It was further tested on the initial online respondents’ data (n = 200) leading to final, minor amendments. The data reported here were collected using a cross-sectional design, although the survey could also be used in a longitudinal design.

#### Arts and cultural activities

Arts and cultural engagement was measured through 20 items, 10 of which asked about participatory engagement and 10 asked about receptive arts engagement. The items were adapted from the list of 21 participatory arts activities and 14 receptive arts activities in *Understanding Society*, predominantly by collapsing the examples across the broader art forms within: literary, visual, performing, crafts and decorative arts. Each item was rated on a seven-point Likert-type scale: 0 = *Never*, 1 = *A one-off engagement*, 2 = *Once or twice a year*, 3 = *Every few months*, 4 = *Monthly*, 5 = *Weekly*, 6 = *Daily*. When considering the scope of arts engagement, we broadened each art form where possible and simultaneously collapsed individual art activity items that appeared to create over-specificity and bias towards a given art form. As a result, each item is inclusive in terms of genre (e.g. we do not exemplify dance with ballet or any other genre), modes of production within the given art form (e.g. singing vs. playing an instrument for making music), and steps in the creative process (e.g. we do not single out practice, rehearsal, or performance). We included an equal number of arts activities within each participatory and receptive engagement (10 each) to ensure a balanced comparison. Finally, to explore whether people engage in the arts in social or isolated ways, we ask whether their engagement is *mainly alone*, *with others or alone*, or *mainly with others*.

Our guidance for respondents’ recall was limited to specifying the broad remit of our arts engagement definitions. Preceding the list of participatory activities, we specified that “This section explores the types of arts and cultural activities that you do as a *participant* as a past-time activity, i.e. where you are actively involved in doing, creating, making, etc. rather than as a spectator, listener, or audience member”. Before the receptive arts engagement activities, we specified that “This section explores the types of past-time arts and cultural activities that you do as a *spectator*, *listener*, *or audience member*, rather than as a participant.” We also included an open-ended question to capture additional arts activities missed in our list of twenty; however, out of 400 responses provided none were clearly separate from the art forms included in our list, and the majority describe non-arts-based leisure activities, such as sports.

#### Demographic information

Demographic information was collected regarding ethnicity, geographic region, educational qualifications, gender, age, living situation, and professional work within the arts. Where possible we used standardized UK Office for National Statistics Census questions (including geographic regions, ethnicities, educational qualifications, and living situations [66]) and attempted to match our sample to the national profile. For further details on the demographic questions and their use in analysis, see S2 File.

A final aspect of demographics eliciting financial information was included as the last part of the survey alongside a question on spending habits on the arts. Household income over the last 12 months from all sources (including all earnings before tax, all pensions and grants, benefits and tax credits, interest from savings or investments, rent from property, and any other income) was based on the Scottish Office of National Statistics survey and was rated on a scale ranging from £5,199 to £76,000 and above. Three newly developed questions related specifically to spending on arts and cultural activities. The first asked participants to estimate how much had been spent on their arts and cultural activities in the last month using a slider that ranged £0-£200. The second asked participants to estimate whether this pattern of spending was likely to go up or down or stay the same in the next 12 months and, if participants did expect a change, the third question asked to explain why they expected the change.

#### Health and social variables

General health was assessed using one item from the Short Form 36 Health Survey [67]. Additionally, diagnosis of mental health problems, cancer, cardiovascular disease, and chronic respiratory disease was assessed. Physical activity was measured using a physical activity scale from the *Whitehall II Study* [68], which measures frequency of engagement in activities mildly, moderately, and vigorously energetically taxing. Frequency is rated on a 4-point scale ranging from “Hardly ever or never” to “3 times a week or more”.

Depressive symptoms were measured using Centre for Epidemiologic Study Depression scale (CES-D) short form. Each of the 8 items is rated with a binary scale (Yes/No) and the number of present depressive symptoms is summed (0–8). A score of three symptoms or more has been commonly used in large scale surveys to indicate depression [3].

Wellbeing in the past month was assessed using the Mental Health Continuum Short Form 14-item scale, which measures wellbeing through hedonic wellbeing and eleven positive functioning dimensions [69]. Each item is rated on a 6-point scale (“never” = 0, “once or twice”, “about once a week”, “2 or 3 times a week”, “almost every day”, “every day” = 5) generating a continuous score ranging from 0–70. Additionally, a categorical variable can be derived denoting: “flourishing mental health” (for responders experiencing at least one of the three hedonic wellbeing and at least six of the eleven positive functioning measures “every day” or “almost every day”), “languishing mental health” (for responders experiencing at least at least one of the three hedonic wellbeing and at least six of the eleven positive functioning measures “never” or “once or twice” in the past month), and “moderate mental health” (for respondents in between the two previous categories).

Loneliness was measured using the scale recommended for large scale surveys and for measuring national levels of loneliness in the UK: UCLA Loneliness Short Form scale [70]. The three-item short-form version (alongside a direct loneliness question) is currently the recommended loneliness measure at the national level in the UK [71]. A fourth item of the scale was also administered, but following Tymoszuk et al. [14], we focused on the three item scale for our analysis. Three items of the scale were rated on a 3-point scale (“hardly ever”, “some of the time”, and “often”) generating a score ranging from 3–9, with 6 as a cut point denoting loneliness cases [72]. Additionally, we used the De Jong Gierveld Loneliness Short Form 6-item scale, which has been also validated for large scale research and distinguishes between emotional and social loneliness [73, 74] in our sensitivity analyses. The global loneliness score ranges from 0–6, with maximum score denoting intense loneliness and both emotional and social loneliness range from 0 to 3, with maximum scores denoting intense emotional or social loneliness [74]. Two additional items assessed the frequency of loneliness (“always”, “often”, “sometimes”, “occasionally”, “hardly ever”, “never”) and intensity of loneliness (“not intense at all”, “a little intense”, “neutral”, “quite intense”, “very intense”). Number of close friends and close family members was assessed with two items rating the number of close persons as “none”, “one”, “two”, “three to four”, “five to eight”, “nine or more”. The presence of a partner and spouse and the quality of that relationship was assessed with one item with the following answer options: “Not applicable, I don’t have a partner or spouse”, “not at all close”, “not very close”, “quite close”, “very close”. Finally, social connectedness was measured with Social Connectedness Revised 15-item scale [75] and the degree of agreement with each question is assessed with a Likert-type scale ranging from “strongly disagree (1)” to “strongly agree (6)” generating a continuous summary score of 15–90. For the ease of interpretation, we scored the scale 0–75.

### Statistical methods

Data were analyzed using Stata V16.1 and a copy of the dataset is publicly available [76]. Descriptive characteristics of the sample and arts engagement can be found in Tables 1 and 2. An arts engagement score (Table 3) was derived by summing the number of arts activities in which the respondent engaged as “one-off” or more frequently, and ranged from 0 to 20 for overall arts engagement score as well as 0–10 each for participatory and receptive arts engagement scores. To identify patterns in arts engagement, the partition cluster-analysis method *kmedians* was used. This clustering method divides the observations into a pre-specified number of distinct, nonoverlapping groups, or clusters. In an iterative process, medians are computed for each cluster center and observations are tested against each cluster to find the closest match until the observation assignment is sustained over two consecutive iterations. To determine the number of clusters, we used the Calinski and Harabasz (1974) pseudo-F index stopping rule [77], which indicated a presence of three distinct clusters in our sample (F = 1181.85). The composition of these clusters in our sample is presented in Table 4. Cluster groupings were tested against Latent Class Analysis findings which also indicated three very similar groupings.

**Table 1:** Demographic, socioeconomic, and self-rated health and fitness characteristics of the sample, UK-wide *HEartS Survey* n = 5,338.

Sociodemographic and Economic Characteristics	
**Age**, mean (SD)	45.96 (16.64)
Age categories, N (%)	
18–25	647 (12.12%)
26–35	1,134 (21.24%)
36–45	945 (17.70%)
46–55	848 (15.89%)
56–65	932 (17.46%)
66–75	707 (13.24%)
76–94	125 (2.34%)
**Gender**, N(%)	
Men	2,636 (49.38%)
Women	2,702 (50.62%)
**Region**, N(%)	
Northern Scotland	120 (2.25%)
Southern Scotland	243 (4.55%)
North East	192 (3.60%)
North West	603 (11.30%)
Yorkshire and the Humber	463 (8.67%)
East Midlands	405 (7.59%)
West Midlands	493 (9.24%)
East of England	505 (9.46%)
South East	742 (13.90%)
South West	475 (8.90%)
London	671 (12.57%)
North Wales	73 (1.37%)
South Wales	206 (3.87%)
Northern Ireland	147 (2.75%)
**Ethnicity**, N(%)	
White British or Irish	4,592 (86.02%)
Any other White background	239 (4.48%)
Mixed ethnic backgrounds*	117 (2.19%)
Asian ethnic backgrounds*	236 (4.42%)
Black ethnic backgrounds*	122 (2.29%)
Any other ethnic background	32 (0.60%)
**Education**, N(%)	
University degree–NVQ Level 4–5	1,974 (36.98%)
A level, baccalaureate–NVQ Level 3	1,193 (22.35%)
GCSE, O Level, AS Level–NVQ Level 1–2	1,353 (25.35%)
Other vocational and foreign qualifications	818 (15.32%)
**Living status**, N(%)	
Lone living	1,122 (21.02%)
With partner only	1,845 (34.56%)
With partner and children	1,317 (24.67%)
With family, house share and other	1,054 (19.75%)
**Household income**, N(%)	
Up to £5,199	228 (4.27%)
£5,200 and up to £10,399	396 (7.42%)
£10,400 and up to £15,599	559 (10.47%)
£15,600 and up to £20,799	606 (11.35%)
£20,800 and up to £25,999	628 (11.76%)
£26,000 and up to £31,199	616 (11.54%)
£31,200 and up to £36,399	398 (7.46%)
£36,400 and up to £41,599	433 (8.11%)
£41,600 and up to £46,799	285 (5.34%)
£46,800 and up to £51,999	306 (5.73%)
£52,000 and up to £75,999	547 (10.25%)
£76,000 and above	336 (6.29%)
**Socioeconomic Aspects of Arts Engagement**	
**Art as profession**, Yes N(%)	664 (12.44%)
**Last month’s spending on art**, mean (SD)	49.08 (47.76)
**Art spending prediction**, N(%)	
To stay the same	3,539 (66.30%)
To increase	1,282 (24.02%)
To decrease	517 (9.69%)
**General Health and Physical Fitness characteristics**	
**Self-rated health**, N(%)	
Very good	808 (15.14%)
Good	2,300 (43.09%)
Fair	1,683 (31.53%)
Bad	415 (7.77%)
Very bad	132 (2.47%)
**Health conditions**, N(%)	
None	3,524 (66.02%)
Mental health problems	1,257 (23.55%)
Cancer	118 (2.21%)
Cardiovascular disease	119 (2.23%)
Chronic respiratory disease	128 (2.40%)
More than one health condition	192 (3.60%)
**Mild physical activity frequency**, N(%)	
Hardly ever or never	975 (18.27%)
About once to 3 times a month	629 (11.78%)
Once or twice a week	1,386 (25.96%)
3 times a week or more	2,348 (43.99%)
**Moderate physical activity frequency**, N(%)	
Hardly ever or never	1,361 (25.50%)
About once to 3 times a month	1,235 (23.14%)
Once or twice a week	1,750 (32.78%)
3 times a week or more	992 (18.58%)
**Vigorous physical activity frequency**, N(%)	
Hardly ever or never	2,861 (53.60%)
About once to 3 times a month	985 (18.45%)
Once or twice a week	920 (17.23%)
3 times a week or more	572 (10.72%)
**Mental Health, Wellbeing and Social characteristics**	
**Depression**–**CES-D 8 score (0–8)**, mean(SD)	3.51 (2.67)
**Depression cases** (≥ 3 score), N(%)	3,045 (57.04%)
**Wellbeing**–**Mental Health Continuum score (0–70)**, mean(SD)	38.42 (16.07)
**Mental Health Continuum categorical variable**, N(%)	
Languishing	862 (16.15%)
Moderate wellbeing	2,792 (52.30%)
Flourishing	1,684 (31.55%)
**Loneliness**–**UCLA score (3–9)**, mean(SD)	5.26 (1.96)
**Loneliness cases** (≥6 score), N(%)	2,448 (45.86%)
**De Jong Gierveld Loneliness Scale (0–6)**, mean(SD)	3.42 (2.01)
Loneliness top score (6 cut point) cases, N(%)	1,137 (21.30%)
Emotional loneliness top score (3 cut point), N(%)	1,652 (30.95%)
Social loneliness top score (3 cut point), N(%)	2,520 (47.21%)
**Frequency of loneliness 1-item**¸N(%)	
Never	728 (13.64%)
Hardly ever	1,227 (22.99%)
Occasionally	930 (17.42%)
Sometimes	1,230 (23.04%)
Often	902 (16.90%)
Always	321 (6.01%)
**Intensity of loneliness 1-item**, N(%)	
Not at all	1,343 (25.16%)
A little	1,553 (29.09%)
Neutral	1,474 (27.61%)
Quite intense	679 (12.72%)
Very intense	289 (5.41%)
**Social connectedness score (0–75)**, mean(SD)	41.48 (15.49)
**Number of close friends**, N(%)	
None	872 (16.34%)
One	738 (13.83%)
Two	1,226 (22.97%)
Three to four	1,711 (32.05%)
Five to eight	588 (11.02%)
Nine or more	203 (3.80%)
**Number of close family members**, N(%)	
None	556 (10.42%)
One	750 (14.05%)
Two	1,128 (21.13%)
Three to four	1,563 (29.28%)
Five to eight	917 (17.18%)
Nine or more	424 (7.94%)
**Closeness with a partner**, N(%)	
No partner/spouse	1,488 (27.88%)
Not at all close	144 (2.70%)
Not very close	362 (6.78%)
Quite close	1,050 (19.67%)
Very close	2,294 (42.97%)

* Ethnicity: Any mixed background includes White and Black Caribbean, White and Black African, White and Asian and any other mixed background; any Asian background includes Indian, Pakistani, Bangladeshi, Chinese or any other Asian background; any Black background includes Caribbean, African, and any other Black background.

**Table 2:** Arts activities listed from most to least popular with their respective frequency of engagement and social context of engagement, UK-wide *HEartS Survey* n = 5,338.

				Frequency of engagement			Social context			
				(Total = Any Engagement + No Engagement)			(Total = Any Engagement)			
	*		Any Engagement**	Daily/Weekly	Monthly/Every few months	One off/Once or twice a year	Mainly with others	Alone and with others	Mainly alone	No Engagement
1	P	Read as a past-time activity	4549 (85.22%)	50.06%	21.86%	13.30%	2.11%	12.0%	85.89%	789 (14.78%)
2	R	Listened to recorded music	4405 (82.52%)	58.30%	16.11%	8.11%	9.08%	40.50%	50.42%	933 (17.48%)
3	R	Watched a film or drama at a cinema	4041 (75.70%)	7.06%	41.12%	27.52%	58.45%	30.44%	11.11%	1297 (24.30%)
4	R	Been to live music	3547 (66.45%)	4.42%	24.75%	37.28%	62.25%	26.81%	10.94%	1791 (33.55%)
5	R	Been to an exhibition, museum etc.	3525 (66.04%)	3.52%	22.86%	39.66%	53.42%	33.16%	13.42%	1813 (33.96%)
6	R	Been to live theatre or circus	3307 (61.95%)	3.30%	17.29%	41.36%	63.26%	27.76%	8.98%	2031 (38.05%)
7	R	Listened to audio books or podcasts	2625 (49.18%)	16.82%	16.65%	15.70%	5.07%	19.35%	75.58%	2713 (50.82%)
8	R	Been to street art, public art displays	2599 (48.69%)	3.18%	16.37%	29.13%	50.17%	35.63%	14.20%	2739 (51.31%)
9	P	Done photography, film, video etc.	2375 (44.49%)	11.05%	18.34%	15.10%	13.52%	39.62%	46.86%	2963 (55.51%)
10	P	Done any crafts or decorative arts	2336 (43.76%)	10.47%	15.89%	17.40%	15.11%	32.32%	52.57%	3002 (56.24%)
11	P	Written as a past-time activity	2339 (43.82%)	10.85%	15.81%	17.16%	3.98%	17.44%	78.58%	2999 (56.18%)
12	P	Played a musical instrument or sang	2197 (41.16%)	15.64%	11.45%	14.07%	17.30%	35.23%	47.47%	3141 (58.84%)
13	R	Been to live dance	2080 (38.97%)	4.11%	11.46%	23.40%	52.50%	33.03%	14.47%	3258 (61.03%)
14	R	Been to crafts or decorative arts fair	2072 (38.82%)	2.87%	11.71%	24.24%	46.48%	37.21%	16.32%	3266 (61.18%)
15	P	Done painting, drawing etc.	2020 (37.84%)	7.94%	14.37%	15.53%	12.82%	30.74%	56.44%	3318 (62.16%)
16	R	Been to a literary event	1702 (31.88%)	3.13%	9.24%	19.52%	28.08%	38.54%	33.37%	3636 (68.12%)
17	P	Practised or performed dance	1402 (26.26%)	5.53%	7.61%	13.13%	32.24%	40.66%	27.10%	3936 (73.74%)
18	P	Attended a book club	1292 (24.20%)	3.48%	9.25%	11.46%	45.36%	31.73%	22.91%	4046 (75.80%)
19	P	Practised or performed a play, drama	1190 (22.29%)	3.32%	5.62%	13.35%	23.19%	38.99%	23.19%	4148 (77.71%)
20	P	Written or created music	1133 (21.23%)	4.93%	7.10%	9.20%	12.36%	33.63%	54.02%	4205 (78.77%)

* P = participatory arts; R = receptive arts

** Total of participants reporting any engagement (*one-off* or more frequently).

**Table 3:** Arts engagement summative scores and other arts engagement-related characteristics of the sample, UK-wide *HEartS Survey* n = 5,338.

**Overall arts engagement**	
Arts engagement summative score (0–20), mean (SD)	9.50 (5.81)
No arts engagement, N(%)	137 (2.57%)
**Participatory arts engagement**	
Participatory arts engagement summative score (0–10), mean (SD)	3.90 (3.12)
No participatory arts engagement, N(%)	502 (9.40%)
**Receptive arts engagement**	
Receptive arts engagement summative score (0–10), mean (SD)	5.60 (3.20)
No receptive arts engagement, N(%)	328 (6.14%)

**Table 4:** Median frequency of engagement for each arts activity per arts engagement cluster, UK-wide *HEartS Survey* n = 5,338.

	Arts engagement clusters		
	“Low engagers”	“Receptive consumers”	“Omnivores”
% of the sample	19.84%	44.38%	35.78%
Read as a past-time activity	*one-off*	*weekly*	*weekly*
Written as a past-time activity	*never*	*never*	*every few months*
Attended a book club	*never*	*never*	*one-off*
Played a musical instrument or sang	*never*	*never*	*every few months*
Written or created music	*never*	*never*	*never*
Practised or performed dance	*never*	*never*	*one-off*
Practised or performed a play, drama	*never*	*never*	*one-off*
Done photography, film, video etc.	*never*	*never*	*every few months*
Done painting, drawing etc.	*never*	*never*	*every few months*
Done any crafts or decorative arts	*never*	*never*	*every few months*
Been to a literary event	*never*	*never*	*once or twice a year*
Listened to audio books or podcasts	*never*	*never*	*monthly*
Been to live music	*never*	*one-off*	*every few months*
Listened to recorded music	*never*	*weekly*	*weekly*
Been to live dance	*never*	*never*	*once or twice a year*
Been to live theatre or circus	*never*	*one-off*	*once or twice a year*
Watched a film or drama at a cinema	*never*	*once or twice a year*	*every few months*
Been to exhibition, museum etc.	*never*	*one-off*	*every few months*
Been to crafts or decorative arts fair	*never*	*never*	*once or twice a year*
Been to street art, public art displays	*never*	*never*	*once or twice a year*

Mean number of arts activities by sociodemographic, socioeconomic, health, and social variables is reported in Table 5 using one-way analysis-of-variance (ANOVA) models for multiple comparisons and Pearson’s chi-squared test for binary variables. Similarly, sociodemographic, socioeconomic, health, and social characteristics were tabulated using Pearon’s chi-squared tests for arts engagement clusters (Table 6). To analyze associations between arts engagement and mental health, social, and wellbeing outcomes, we used regression analyses. Using logistic regression, we investigated the odds of scoring as cases on depressive symptoms (CES-D ≥3) and loneliness (R-UCLA ≥6) for the three arts engagement scores (overall, participatory, and receptive; Table 7) and for the three arts engagement clusters using *low engagers* as a reference category (Table 8). We adjusted the associations in both logistic and linear regression models for sociodemographic factors: gender, age, ethnicity (Model 1); indicators of socioeconomic status: educational attainment and household income (Model 2); self-rated health and mild, moderate, and vigorous physical activity engagement (Model 3); and finally, living status and closeness of relationship with a partner (Model 4). Linear regression models were used to compare levels of wellbeing and social connectedness across three arts engagement scores (overall, participatory, and receptive, Table 7) and the three arts engagement clusters using the *low engagers* as a reference category (Table 8) and the same covariate adjustment strategy as in logistic regression models. Ordinary least squares regression assumptions were checked using well-established tests: the assumption of normality of residuals was tested using kernel density plot, standardized normal probability (P-P) plot, and Q-Q plots, while homoscedasticity of residuals was established by plotting residuals versus predicted values and White’s test. These tests indicated significant departures from normality and homoscedasticity, hence the (robust) sandwich variance estimator was imposed to correct for non-constant residual variance. Multicollinearity was not detected using variance inflation factor, and hence no redundant covariates were identified.

**Table 5:** Mean number of arts activities by sociodemographic, socioeconomic, health, and social variables from ANOVA models and Pearson’s chi-squared test, UK-wide *HEartS Survey* n = 5,338.

	Overall arts score	Participatory arts score	Receptive arts score
	mean (SD)	mean (SD)	mean (SD)
**Age categories**			
18–25 (ref)	13.33 (5.68)	6.42 (3.11)	6.91 (2.99)
26–35	11.79 (6.07)**	5.29 (3.28))**	6.50 (3.19)
36–45	10.18 (5.85))**	4.16 (3.16))**	6.02 (3.20))**
46–55	8.22 (5.17))**	3.14 (2.60))**	5.08 (3.10))**
56–65	7.12 (4.47))**	2.41 (2.06))**	4.71 (2.96))**
66–75	6.75 (4.21))**	2.23 (1.88))**	4.51 (2.87))**
76–94	5.93 (4.08))**	2.12 (1.89))**	3.81 (2.81))**
**Gender**			
Women	9.71 (5.63)	4.08 (3.22)	5.64 (3.15)
Men	9.29 (5.98))**	3.72 (3.00))**	5.57 (3.24)
**Ethnicity**			
Black Asian and minority ethnicities	12.29 (6.32)	5.64 (3.46)	6.65 (3.30)
White British and other	9.21 (5.68))**	3.72 (3.02))**	5.49 (3.16))**
**Education**^a^			
University degree–NVQ Level 4–5 (ref)	11.36 (5.75)	4.82 (3.19)	6.54 (3.04)
A level, baccalaureate–NVQ Level 3	9.41 (5.48))**	3.87 (3.00))**	5.55 (3.06))**
GCSE, O Level, AS Level–NVQ Level 1–2	8.32 (5.83))**	3.37 (3.09))**	4.95 (3.23))**
Other vocational and foreign qualifications	7.11 (4.92))**	2.63 (2.39))**	4.48 (3.06))**
**Region**			
England (ref)	9.50 (5.84)	3.91 (3.14)	5.59 (3.20)
Scotland	10.12 (5.59)	4.14 (3.01)	5.98 (3.14)
Wales	8.97 (5.81)	3.65 (3.02)	5.32 (3.26)
Northern Ireland	9.28 (5.36)	3.59 (2.86)	5.69 (3.12)
**Household income**			
Above median	10.05 (5.65)	4.02 (3.09)	6.03 (3.06)
Below median	8.84 (5.94))**	3.76 (3.14))**	5.08 (3.28))**
**Art as profession**			
Yes	16.23 (4.53)	7.79 (2.58)	8.44 (2.33)
No	8.55 (5.32))**	3.35 (2.77))**	5.20 (3.09))**
**Art spending prediction**			
To stay the same (ref)	8.46 (5.51)	3.36 (2.87)	5.10 (3.14)
To increase	12.42 (5.71))**	5.40 (3.31))**	7.01 (2.93))**
To decrease	9.44 (5.64))**	3.92 (2.99))**	5.52 (3.16)*
**Self-rated health**			
Very good / good (ref)	10.10 (5.91)	4.14 (3.22)	5.96 (3.17)
Fair	8.82 (5.56))**	3.59 (2.95))**	5.23 (3.16))**
Bad / very bad	8.24 (5.56))**	3.53 (2.90))**	4.72 (3.14))**
**Mild physical activity frequency**			
Hardly ever or never (ref)	6.34 (5.18)	2.55 (2.66)	3.79 (3.07)
Monthly and weekly engagement	10.21 (5.71))**	4.20 (3.13))**	6.01 (3.08))**
**Moderate physical activity frequency**			
Hardly ever or never (ref)	6.27 (4.86)	2.50 (2.46)	3.77 (2.95)
Monthly and weekly engagement	10.61 (5.70))**	4.38 (3.17))**	6.23 (3.03))**
**Vigorous physical activity frequency**			
Hardly ever or never (ref)	7.37 (4.85)	2.85 (2.45)	4.52 (2.96)
Monthly and weekly engagement	11.97 (5.86))**	5.12 (3.35))**	6.85 (2.99))**
**Living situation**			
Lone living (ref)	8.76 (5.82)	3.56 (3.07)	5.20 (3.28)
With partner only	8.62 (5.37)	3.29 (2.82)	5.34 (3.09)
With partner and children	10.47 (6.00))**	4.38 (3.24))**	6.09 (3.22))**
With family, house share and other	10.63 (5.94))**	4.75 (3.21))**	5.89 (3.18))**
**Closeness with a partner**			
No partner/spouse (ref)	8.54 (5.60)	3.55 (2.92)	4.99 (3.18)
Not at all or very close	11.24 (6.52))**	4.94 (3.55))**	6.30 (3.41))**
Quite or very close	9.67 (5.71))**	3.90 (3.09))**	5.77 (3.13))**
**Depression cases**			
Cases (≥ 3 score, ref)	10.10 (5.97)	4.32 (3.20)	5.78 (3.24)
Non cases	8.73 (5.49))**	3.36 (2.90))**	5.37 (3.12))**
**Wellbeing categorical variable**			
Languishing (ref)	7.72 (5.32)	3.29 (2.74)	4.43 (3.10)
Moderate wellbeing	9.83 (5.82))**	4.03 (3.18))**	5.80 (3.15))**
Flourishing	9.87 (5.86))**	4.01 (3.17))**	5.87 (3.18))**
**Loneliness** (UCLA scale)	9.97 (5.92)	4.27 (3.17)	5.70 (3.24)
Cases (score ≥ 6 score, ref)			
Non cases	9.11 (5.69))**	3.59 (3.04))**	5.52 (3.15)*
**Loneliness** (De Jong Gierveld scale)			
Cases (score = 6, ref)	9.90 (6.01)	4.31 (3.19)	5.59 (3.32)
Non cases	9.40 (5.75))**	3.79 (3.09))**	5.61 (3.16)
**Social connectedness**			
Above the median (ref)	9.00 (5.34)	3.42 (2.82)	5.57 (3.04)
Below the median	10.00 (6.20))**	4.37 (3.31))**	5.63 (3.34)

* p<0.*05;*

** p<0.*01*.

^a^ Full list of categories listed in Methods

^b^ Household income median = £26,000-£31,199.

**Table 6:** Sociodemographic, health, and social characteristics of each cluster of arts activities using Pearson’s chi-squared test, UK-wide *HEartS Survey* n = 5,338.

	“Low engagers”	“Receptive consumers”	“Omnivores”	*p*
	N = 1,058 (19.8%)		N = 1,909 (35.8%)	
		N = 2,371 (44.4%)		
**Age**, mean (SD)	49.69 (16.50)	50.98 (15.76)	37.67 (14.40)	*<0*.*001*
**Gender**, women N(%)	526 (49.67%)	1,124 (47.45%)	1,052 (55.08%)	*<0*.*001*
**Ethnicity**, ethnic minorities N(%)	105 (9.92%)	134 (5.66%)	268 (14.03%)	*<0*.*001*
**Education**^a^, N(%)				
University degree–NVQ Level 4–5	256 (24.17%)	735 (31.03%)	983 (51.47%)	*<0*.*001*
A level, baccalaureate–NVQ Level 3	203 (19.17%)	581 (24.53%)	409 (21.41%)	
GCSE, O Level, AS Level–NVQ Level 1–2	369 (34.84%)	614 (25.92%)	370 (19.37%)	
Other vocational and foreign qualifications	231 (21.81%)	439 (18.53%)	148 (7.75%)	
**Region**, N(%)				
England	924 (87.25%)	1,999 (84.38%)	1,626 (85.13%)	*0*.*008*
Scotland	44 (4.15%)	173 (7.30%)	146 (7.64%)	
Wales	61 (5.76%)	125 (5.28%)	93 (4.87%)	
Northern Ireland	30 (2.83%)	72 (3.04%)	45 (2.36%)	
**Household income** ≥median^b^, N(%)	473 (44.66%)	1,318 (55.64%)	1,130 (59.26%)	*<0*.*001*
**Art as profession**, Yes N(%)	33 (3.12%)	62 (2.62%)	569 (29.79%)	*<0*.*001*
**Last month’s spending on art**, mean (SD)	27.20 (39.37)	39.86 (41.59)	72.7 (49.45)	*<0*.*001*
**Art spending prediction**, N(%)				
To stay the same	772 (72.90%)	1,753 (74.00%)	1,014 (53.09%)	*<0*.*001*
To increase	156 (14.73%)	413 (17.43%)	713 (37.33%)	
To decrease	131 (12.37%)	203 (8.57%)	183 (9.58%)	
**Self-rated health,** N(%)				
Very good / good	540 (50.99%)	1,362 (57.49%)	1,206 (63.14%)	*<0*.*001*
Fair	379 (35.79)	772 (32.59%)	532 (27.85%)	
Bad / very bad	140 (13.22%)	235 (9.92%)	172 (9.01%)	
**Mild physical activity frequency**				
Hardly ever or never vs. monthly or weekly	686 (64.78%)	1,939 (81.85%)	1,738 (90.99%)	*<0*.*001*
**Moderate physical activity frequency**				
Hardly ever or never vs. monthly or weekly	581 (54.86%)	1,731 (73.07%)	1,665 (87.17%)	*<0*.*001*
**Vigorous physical activity frequency**				
Hardly ever or never vs. monthly or weekly	343 (32.39%)	865 (36.51%)	1,269 (66.44%)	*<0*.*001*
**Living situation**				
Lone living	243 (22.95%)	539 (22.75%)	340 (17.80%)	*<0*.*001*
With partner only	403 (38.05%)	921 (38.88%)	521 (27.28%)	
With partner and children	238 (22.47%)	515 (21.74%)	564 (29.53%)	
With family, house share and other	175 (16.53%)	394 (16.63%)	485 (25.39%)	
**Closeness with a partner**				
No partner/spouse	319 (30.12%)	701 (29.59%)	468 (24.50%)	*<0*.*001*
Not at all or very close	104 (9.82%)	173 (7.30%)	229 (11.99%)	
Quite or very close	636 (60.06%)	1,495 (63.11%)	1,213 (63.51%)	
**Depression cases**				
Cases (≥ 3 score) vs. non-cases	604 (57.03%)	1,201 (50.70%)	1,240 (64.92%)	*<0*.*001*
**Wellbeing categorical variable**				
Languishing	269 (25.40%)	369 (15.58%)	224 (11.73%)	*<0*.*001*
Moderate wellbeing	501 (47.31%)	1,254 (52.93%)	1,037 (54.29%)	
Flourishing	289 (27.29%)	746 (31.49%)	1,037 (54.29%)	
**Loneliness** (UCLA scale)	486 (45.89%)	1,003 (42.34%)	959 (50.21%)	*<0*.*001*
Cases (score ≥ 6 score) vs. non-cases				
**Loneliness** (De Jong Gierveld scale)				
Cases (score = 6) vs. non-cases	263 (24.83%)	435 (18.36%)	439 (22.98%)	*<0*.*001*
**Social connectedness**				
Above the median vs. below	483 (45.61%)	1,322 (55.80%)	848 (44.40%)	*<0*.*001*

^a^ Full list of categories listed in S2 File

^b^ Household income median = £26,000-£31,199.

**Table 7:** Results from logistic regression analyses examining the association between arts engagement score and odds of depression and loneliness as well as linear regression analyses examining the association between arts engagement score, wellbeing, and social connectedness. All arts engagement variables and all outcomes were entered and analysed in individual models, UK-wide *HEartS Survey* n = 5,338.

	Depression				UCLA loneliness				Wellbeing				Social Connectedness			
	OR	95% CI	*p*	R^2^	OR	95% CI	*p*	R^2^	B	95% CI	*p*	R^2^	B	95% CI	*p*	R^2^
Model 1: sociodemographic variables																
*Overall arts activities score*	1.01	1.00–1.02	*0*.*15*	5.56%	0.99	0.98–1.00	*0*.*10*	4.44%	0.67	0.59–0.75	*<0*.*001*	7.26%	0.24	0.16–0.31	*<0*.*001*	7.66%
*Participatory arts activities score*	1.04	1.01–1.06	*0*.*001*	5.69%	1.00	0.98–1.02	*0*.*98*	4.41%	1.00	0.85–1.17	*<0*.*001*	5.27%	0.10	-0.03–0.24	*0*.*14*	7.05%
*Receptive arts activities score*	1.00	0.98–1.01	*0*.*63*	5.54%	0.97	0.96–0.99	*0*.*004*	4.52%	1.21	1.07–1.36	*<0*.*001*	7.64%	0.63	0.50–0.75	*<0*.*001*	8.54%
Model 2: Model 1 + socioeconomic variables																
*Overall arts activities score*	1.02	1.01–1.03	*0*.*001*	7.71%	1.00	0.99–1.01	*0*.*48*	5.76%	0.61	0.53–0.69	*<0*.*001*	9.48%	0.18	0.11–0.26	*<0*.*001*	10.47%
*Participatory arts activities score*	1.05	1.02–1.07	*<0*.*001*	7.82%	1.00	0.98–1.02	*0*.*77*	5.75%	0.93	0.78–1.09	*<0*.*001*	8.08%	0.06	-0.08–0.20	*0*.*39*	10.10%
*Receptive arts activities score*	1.02	1.00–1.04	*0*.*08*	7.61%	0.99	0.97–1.00	*0*.*13*	5.79%	1.08	0.93–1.22	*<0*.*001*	9.57%	0.51	0.38–0.64	*<0*.*001*	11.04%
Model 3: Model 2 + health and fitness variables																
*Overall arts activities score*	1.03	1.02–1.05	*<0*.*001*	15.56%	1.01	0.99–1.02	*0*.*34*	9.94%	0.42	0.34–0.50	*<0*.*001*	23.42%	0.04	-0.04–0.11	*0*.*34*	20.51%
*Participatory arts activities score*	1.07	1.04–1.09	*<0*.*001*	15.59%	1.01	0.99–1.04	*0*.*19*	9.95%	0.65	0.50–0.80	*<0*.*001*	22.91%	-0.16	-0.29–0.02	*0*.*025*	20.57%
*Receptive arts activities score*	1.05	1.02–1.07	*<0*.*001*	15.40%	1.00	0.98–1.03	*0*.*66*	9.93%	0.71	0.56–0.85	*<0*.*001*	23.32%	0.24	0.11–0.38	*<0*.*001*	20.69%
Model 4: Model 3 + social circumstances variables																
*Overall arts activities score*	1.03	1.02–1.05	*<0*.*001*	18.94%	1.00	0.99–1.02	*0*.*56*	16.08%	0.45	0.37–0.53	*<0*.*001*	28.81%	0.07	-0.004–0.14	*0*.*06*	26.29%
*Participatory arts activities score*	1.06	1.03–1.09	*<0*.*001*	18.95%	1.01	0.98–1.03	*0*.*54*	16.08%	0.72	0.57–0.87	*<0*.*001*	28.34%	-0.08	-0.21–0.06	*0*.*26*	26.26%
*Receptive arts activities score*	1.04	1.02–1.07	*<0*.*001*	18.83%	1.01	0.98–1.03	*0*.*65*	16.08%	0.72	0.58–0.86	*<0*.*001*	28.58%	0.27	0.14–0.40	*<0*.*001*	26.48%

Abbreviations: OR, odds ratio; CI, confidence interval; B, regression coefficient; R^2^, Pseudo R-squared in logistic regression models and R-squared in linear regression models.

Cut points for cases: Depression CES-D ≥3; loneliness R-UCLA ≥6.

Model 1: Gender, age, ethnicity; Model 2: Model 1 + educational attainment, household income; Model 3: Model 2 + self-rated health and physical activity (mild, moderate, vigorous); Model 4: Model 3 + living status, closeness of relationship with a partner.

**Table 8:** Results from logistic regression analyses examining the association between arts engagement clusters and odds of depression and loneliness as well as linear regression analyses examining the association between arts engagement clusters, wellbeing, and social connectedness. All outcomes were entered and analysed in individual models, UK-wide *HEartS Survey* n = 5,338.

	Depression				UCLA loneliness					Wellbeing				Social Connectedness			
	OR	95% CI	*p*	R^2^	OR	95% CI	*p*	R^2^	B		95% CI	*p*	R^2^	B	95% CI	*p*	R^2^
Model 1: sociodemographic variables (reference: low engagers)																	
*Receptive consumers*	0.79	0.68–0.92	*0*.*002*	5.72%	0.90	0.77–1.05	*0*.*18*	4.48%	4.20		2.96–5.43	*<0*.*001*	6.30%	2.90	1.79–4.02	*<0*.*001*	*7*.*80%*
*Omnivores*	0.96	0.82–1.14	*0*.*68*		0.82	0.70–0.97	*0*.*020*		9.16		7.85–10.48	*<0*.*001*		3.91	2.76–5.07	*<0*.*001*	
Model 2: Model 1 + socioeconomic variables (reference: low engagers)																	
*Receptive consumers*	0.86	0.74–1.01	*0*.*06*	7.76%	0.95	0.82–1.11	*0*.*54*	5.78%	3.56		2.34–4.79	*<0*.*001*	8.65%	2.33	1.22–3.44	*<0*.*001*	*10*.*58%*
*Omnivores*	1.11	0.94–1.32	*0*.*22*		0.89	0.75–1.05	*0*.*17*		8.16		6.85–9.48	*<0*.*001*		3.12	1.96–4.29	*<0*.*001*	
Model 3: Model 2 + health and fitness variables (reference: low engagers)																	
*Receptive consumers*	0.94	0.80–1.12	*0*.*66*	15.40%	1.04	0.89–1.23	*0*.*59*	9.94%	2.31		1.15–3.48	*<0*.*001*	23.33%	1.31	0.23–2.39	*0*.*018*	*20*.*62%*
*Omnivores*	1.29	1.06–1.56	*0*.*005*		0.99	0.83–1.19	*0*.*94*		5.87		4.59–7.16	*<0*.*001*		1.49	0.33–2.65	*0*.*012*	
Model 4: Model 3 + social circumstances variables (reference: low engagers)																	
*Receptive consumers*	0.96	0.81–1.14	*0*.*67*	18.84%	1.07	0.91–1.27	*0*.*43*	16.09%	2.18		1.06–3.30	*<0*.*001*	28.52%	1.11	0.07–2.14	*0*.*036*	*26*.*34%*
*Omnivores*	1.30	1.07–1.57	*0*.*009*		1.01	0.84–1.22	*0*.*89*		5.80		4.57–7.04	*<0*.*001*		1.42	0.30–2.54	*0*.*013*	

Abbreviations: OR, odds ratio; CI, confidence interval; B, regression coefficient; R^2^, Pseudo R-squared in logistic regression models and R-squared in linear regression models.

Cut points for cases: Depression CES-D ≥3; loneliness R-UCLA ≥6.

Model 1: Gender, age, ethnicity; Model 2: Model 1 + educational attainment, household income; Model 3: Model 2 + self-rated health and physical activity (mild, moderate, vigorous); Model 4: Model 3 + living status, closeness of relationship with a partner.

We conducted additional sensitivity analyses on the association between arts engagement and loneliness using the De Jong Gierveld loneliness scale which enables investigation of both global loneliness as well as its emotional and social dimensions [74]. Using logistic regression, we investigated the odds of maximum scores of 6 denoting intense global loneliness as well as maximum scores of 3 denoting intense emotional or social loneliness [74] for the three arts engagement scores (overall, participatory, and receptive–S1 Table–and the three arts engagement clusters using the *low engagers* as a reference category–S2 Table).

## Results

### Sample description

Descriptive characteristics of the sample are reported in Table 1. The mean age was 45.96 years, women constituted 50.62% of the respondents, and the sample was predominantly White British or Irish (86.02%), mirroring the overall gender and ethnic distribution in the UK. The majority of the respondents reported residing in England (85.22%), followed by 6.80% in Scotland, 5.23% in Wales, and 2.75% in Northern Ireland. Over a third of respondents (36.98%) reported their highest educational attainment as University degree or equivalent; 22.35% reported A Level, baccalaureate, or equivalent; 25.35% reported GCSE or equivalent; and the remaining 15.32% reported other vocational qualifications such as youth training certification and foreign qualifications.

Approximately one in five respondents (21.02%) reported living on their own, 34.56% reported living only with a partner, 24.67% reported living with partner and children, and the remaining 19.75% lived with extended family, in house shares, or other arrangements including residential living facilities. Overall household income from all sources over 12 months was evenly distributed between £5,199 to £76,000 and above, with the sample median being £26,000–£31,199. 12.44% of respondents reported working as a professional artist, performer, or maker. The mean spending on art from respondents’ last month was £49.08 (SD £47.76), and 66.30% respondents predicted that their spending on art will remain the same in the future, 24.02% reported an expected increase and 9.69% reported an expected decrease in spending.

Over 40% of the sample reported good health (43.09%), followed by 31.53% rating their health as “fair”, 15.14% rating their health as “very good”, 7.77% rating their health as “bad”, and 2.47% rating their health “very bad”. The majority of the sample did not report any health problems (66.02%), 23.55% reported having mental health problems, 2.21% reported cancer, 2.23% reported cardiovascular disease, 2.40% reported chronic respiratory disease, and 3.60% reported more than one health condition. The majority of respondents reported engaging in mild physical activity three times a week or more often (43.99%), in moderate physical activity once or twice a week (32.78%), and in vigorous physical activity hardly ever or never (53.60%).

Over 57% of respondents scored as “depression cases” having reported three or more depressive symptoms on the CES-D scale. Despite the majority of the sample reporting high number of depressive symptoms, only 16.15% of respondents reported low or “languishing” wellbeing and the majority reported moderate wellbeing (52.30%) and “flourishing” (31.55%). Over 45% of the sample reported loneliness using the 6 cut-point on the UCLA short form scale, and 21.30% reported the maximum score on the De Jong Gierveld Loneliness short form scale indicating severe loneliness. Looking at emotional and social loneliness subsections of the De Jong Gierveld Loneliness scale, roughly 30% reported maximum emotional loneliness score, and over 47% reported maximum social loneliness score. Furthermore, 22.91% reported experiencing loneliness as frequently as “often” and “always”, and a further 18.13% reported their experienced loneliness as “quite” and “very” intense. Despite high levels of loneliness in the sample, the respondents were relatively socially connected both in terms of: (1) structural or quantitative indices–i.e. 83.66% had at least one close friend, 89.58% had at least one close family member, and 72.12% had a partner or a spouse, and (2) functional or qualitative aspects–i.e. average score of social connectedness was 41.48 (SD 15.49) out of 75 and the majority of participants with partners and spouses reported having a close relationship (86.86% out of 3,850).

### Patterns of arts engagement in the United Kingdom 2018–2019

#### Frequency of engagement and popularity of arts activities

All arts activities, listed in descending order of popularity, can be found in Table 2. The most popular activities were those that involve engaging in art that has already been created (watching, listening, reading) rather than creating new art (writing, playing music, acting, dancing). Indeed, the eight most popular activities involve consuming art, for example by reading, listening to music, watching a film or drama. The top six most popular activities were reported by more than 50% of the sample, ranging from 85.22% for reading to 61.95% for visits to live theater or circus (Table 2). The art form itself was not a useful predictor of popularity of engagement. For instance, the same art (music) was engaged with as one of the most and least popular activities: 82.52% listened to recorded music, 66.45% had been to live music, 41.16% played a musical instrument or sang, and only 21.23% had written or created music (Table 2).

The two most popular activities, reading and listening to recorded music, were also most likely to be engaged with the most frequently, on a *daily/weekly* basis by 50.06% and 58.30% of respondents respectively. Several receptive arts activities, although popular, were mainly engaged in infrequently. For example, over a third of those who had been to live music, an exhibition, museum etc. or to live theater or circus did so as a *one-off* or *once or twice a year* (37.28%, 39.66%, and 41.36% respectively). The less popular activities were, on the whole, also engaged in less frequently; for example, the largest proportion of those who had been to live dance, crafts or decorative arts fairs, practiced or performed a play or drama, or written or created music did so as a *one-off* or *once or twice a year*. For some arts activities, engagement frequency was split evenly across the rating scale; for instance, *daily/weekly*, *monthly/every few months*, and *one-off/once or twice a year* engagement was reported, respectively, by 16.82%, 16.65%, 15.70% of respondents listening to audiobooks and podcasts, and 15.64%, 11.45%, 14.07% of respondents playing a musical instrument or singing.

#### Social context

Predominantly solitary activities include literary arts (Table 2), including reading (85.89% engaged mainly alone), writing (78.58% engaged mainly alone), listening to audiobooks or podcasts (75.58% engaged mainly alone), participatory visual arts such as painting or drawing (56.44% engaged mainly alone), and crafts and decorative arts (52.57% engaged mainly alone). Predominantly social activities include popular receptive arts activities: live theater or circus (63.26% engaged mainly with others), live music concerts (62.25% engaged mainly with others), watching film screenings (58.45% engaged mainly with others), visiting an exhibition or museums (53.42% engaged mainly with others), and less popular live dance (52.50% engaged mainly with others). Looking at pooled results for participatory arts engagement, 56.8% of the engagement occurred *mainly alone*, 28.11% *alone and with others*, and 15.09% *mainly with others*. Considering pooled results for receptive arts engagement, 43.19% of the engagement occurred *mainly with others*, 32.10% *alone and with others*, and 24.71% *mainly alone*.

#### Comparing two common approaches of operationalizing arts engagement in quantitative research

Using a summative arts activity score of total number of activities engaged as *one-off* or more frequently, we found that over 97% our sample reported engaging with at least one participatory or receptive arts activity in the last 12 months (Table 3). On average, respondents reported just under half of the arts activities suggested in the survey (mean = 9.50 activities out of 20) and engaged in more receptive arts activities (mean = 5.60 out of 10) than participatory ones (mean 3.90 out of 10). Using the clusters approach to explore profiles of arts engagement in the sample, three distinct clusters were identified: cluster 1 “*low engagers*”, cluster 2 “*receptive consumers*”, and cluster 3 “*omnivores*”, which respectively constituted 19.8%, 44.4%, and 35.8% of the sample (Table 4). *Low engagers* include participants who read infrequently (*one-off* or *once or twice a year*). The *receptive consumers* include those who, in addition to being frequent readers, participate in a range of receptive activities (frequently listening to recorded music, less frequently going to the cinema, and least frequently going to live music, to live theater or circus, and to exhibitions or museum etc.). The *omnivores* include people who participate in almost all the activities with a varying degree of frequency (Table 4).

### Demographic, socioeconomic, health, and social characteristics of arts engagement

#### Summative arts engagement score

The mean number of arts activities (overall, participatory, and receptive) by demographic, socioeconomic, health, and social variables is presented in Table 5. Overall, respondents who had higher arts engagement scores were more likely to be younger (18–25 years of age), women, with higher education attainment, higher income, and belong to ethnic minorities. (The exception to this pattern was that there was no difference between men and women on the receptive arts score.) Respondents who had higher overall, participatory, and receptive arts engagement scores also reported better health, more frequent physical activity, and were more likely to live in bigger households (i.e. with children and spouses as well as extended family or house shares). Not having a partner or spouse was associated with fewer overall, participatory, and receptive arts activities compared with having a coupled relationship, regardless of its closeness. The largest difference in the number of arts activities was between respondents who work in the arts and those who do not (overall 16.23 vs. 8.55, participatory 7.79 vs. 3.35, receptive 8.44 vs. 5.20). Respondents who predicted their art spending to stay the same in the next 12 months reported fewer arts activities relative to those who reported an expected increase or decrease.

In terms of wellbeing measures, respondents scoring as depression and loneliness cases reported more arts activities. (The exception to this was the scores on the De Jong Gierveld loneliness scale where those scoring as lonely and those scoring as not lonely did not have different receptive engagement scores.) Reporting lower levels of social connectedness was also associated with reporting more arts activities overall and more participatory arts activities, but not receptive arts activities. Conversely, moderate and flourishing wellbeing were associated with higher number of overall, participatory, and receptive arts activities compared with languishing.

#### Arts engagement clusters

The distribution of demographic, socioeconomic, health, and social characteristics for three arts engagement clusters *low engagers*, *receptive consumers*, and *omnivores* is presented in Table 6. In line with the findings based on the summative arts engagement score, greater arts engagement, here captured in *omnivores*, is associated with younger age, women, ethnic minorities, higher educational attainment, and income compared with the other two clusters *low engagers* and *receptive consumers* which capture respondents engaging with the arts to a lesser extent. Similarly, compared with *low engagers* and *receptive consumers*, *omnivores* were more likely to rate their health as very good or good, report more mild, moderate, and vigorous physical activity, and live with extended family, house share, and other living arrangements rather than with partner only or alone. They were also less likely to report not having a partner or a spouse. *Omnivores* were also significantly more likely to work in the arts (29.79% vs. 3.12% of *low engagers*) and spend more on arts in a month (£72.7 vs. £27.20 for *low engagers* and £39.86 for *receptive consumers*).

Broadly in line with the findings based on the summative arts engagement score, compared with *low engagers* and *receptive consumers*, highly engaged *omnivores* were more likely to score as depression cases (64.92% vs. 50.70% of *receptive consumers)* and loneliness cases using the UCLA scale (50.21% vs. 42.35% of *receptive consumers*). *Omnivores* were less likely to be “languishing” and more likely to be “flourishing” compared with *low engagers* and *receptive consumers* (Table 6). Unlike the findings based on the summative arts engagement score, using the De Jong Gierveld scale and intense loneliness cut off, *low engagers* were most likely to be intensely lonely (24.83%), followed by *omnivores* (22.98%) and *receptive consumers* (18.38%). Additionally, *receptive consumers* were also more likely to score above the median of social connectedness compared with the other two groups.

*Low engagers* and *receptive consumers* appear to be very similar across the majority of the demographic, socioeconomic, health, and social characteristics (Table 6). They mainly differ in education (*low engagers* tend to be less educated than *receptive consumers*), household income (*low engagers* tend to have lower household income than *receptive consumers*), general health and fitness (*low engagers* tend to be less likely to report very good health and mild/moderate physical activity than *receptive consumers*), depression (57.03% of *low engagers* were depression cases compared with 50.70% of *receptive consumers*), and social connectedness (*low engagers* tend to score lower social connectedness than *receptive consumers*).

### Associations of arts engagement with positive and negative aspects of mental and social wellbeing

In this final section, using regression modelling we explore how the current patterns of arts engagement (operationalized as both summative arts engagement scores and arts engagement clusters) correlate with positive and negative aspects of mental wellbeing (captured through depressive symptoms and wellbeing) as well as positive and negative aspects of social wellbeing (captured through loneliness and social connectedness).

#### Depression

Using a cut-point of three or more to denote depressive symptoms cases (n = 3,045, 57.04%), we found similar patterns of associations for both summative arts activities scores and art clusters in the regression models. Looking at summative arts activities scores, the participatory arts activities score was most strongly associated with odds of depression before and after covariate adjustments (in the final fully adjusted model, odds of depression increased 1.07 with each participatory activity reported). The associations between overall or receptive arts activities score and odds of depression became statistically significant after accounting for socioeconomic variables (Table 7).

Results from models including arts engagement clusters are widely in agreement (Table 8) showing that, compared with *low engagers*, *omnivores* had higher odds of depressive symptoms once health and fitness factors are adjusted for, and this difference remained in the final model adjusted for social circumstances. Additionally, *receptive consumers* appeared to have lower odds of reporting depressive symptoms compared with *low engagers* in the model adjusting for demographic factors (Model 1); however, once socioeconomic, health, and fitness variables were additionally accounted for the difference was no longer statistically significant. The fully adjusted models for summative arts activity score and arts engagement clusters accounted for approximately 19% variability in the outcome.

#### Wellbeing

We found a positive association between arts engagement and wellbeing consistent across both measurement approaches. Overall, participatory and receptive arts engagement scores were positively associated with wellbeing, with marginally larger effect size for receptive arts score before adjustment for health and fitness variables and social circumstances in particular (Table 7).

In terms of the clusters, across all step-wise covariate-adjustment models, both *receptive consumers* and *omnivores* reported higher wellbeing compared with *low engagers*, before and after covariate adjustments, with the effect size for *omnivores* being consistently twice the size of *receptive consumers* (e.g. final model 5.80 vs. 2.18, Table 8). The fully adjusted model for summative arts activity score and arts engagement clusters accounted for approximately 28% variability in the outcome.

#### Loneliness

Using the UCLA loneliness scale and the threshold of six or more for loneliness cases (n = 2,448, 45.86%), we found no consistent association between arts engagement and loneliness for both summative arts activities scores and art clusters. Our sensitivity analyses using the De Jong Gierveld scale and the threshold of 6 for intense global loneliness (n = 1,137, 21.30%) also found no consistent associations between intense loneliness and summative overall, participatory, and receptive arts engagement scores (S1 Table). However, both *receptive consumers* and *omnivores* had lower odds of intense loneliness compared with *low engagers*, before and after covariate adjustments (S2 Table). The fully adjusted model accounted for 11% variability in the outcome (R^2^ = 0.114).

Looking separately at emotional and social dimensions of De Jong Gierveld loneliness scale, we found that overall, participatory, and receptive arts engagement scores were positively associated with intense emotional loneliness (n = 1,652, 30.95%), with marginally larger effect size for participatory arts score (S1 Table). Contrary to this, overall, participatory, and receptive arts engagement scores were negatively associated with intense social loneliness (n = 2,520, 47.21%), with marginally larger magnitude of the association for receptive arts scores (S1 Table). Similarly, in terms of the clusters, compared with *low engagers*, both *receptive consumers* and *omnivores* had lower odds of intense social loneliness. Unlike in models with summative arts engagement scores, in arts engagement cluster models, *receptive consumers* had lower odds of intense emotional loneliness compared with *low engagers* before and after all adjustments, and there was no difference between *omnivores* and *low engagers* (S2 Table). The fully adjusted models accounted for approx. 8% variability in intense social loneliness and between approximately 14% in intense emotional loneliness.

#### Social connectedness

We found a somewhat consistently positive association between arts engagement and social connectedness across both measurement approaches. Using summative arts engagement scores, only receptive arts engagement was consistently positively associated with social connectedness (Table 7).

Using art clusters, across all stepwise covariate-adjustment models, both *receptive consumers* and *omnivores* showed higher social connectedness compared with *low engagers*, before and after covariate adjustments; however, adjustment for health and fitness variables significantly attenuated the effect sizes (Table 8). The final, fully adjusted art clusters regression model explained approximately 26% of variability in social connectedness outcome.

## Discussion

This study has investigated common approaches to the conceptualization and measurement of arts engagement at the population level, in particular for quantitative research seeking to understand arts engagement in the context of population health and wellbeing. We explored patterns and the social context of arts engagement during 2018–2019 using an online sample aligned with the overall distributions of key sociodemographic characteristics (gender, age, geographical region, ethnicity, and education) of the United Kingdom (UK) population. We further investigated the associations between arts engagement and both positive and negative aspects of social and mental wellbeing.

We found that the most popular arts activities, engaged with at least once a year by more than 50% of the sample, involved consuming art (reading or listening to recorded music) that has been already created, rather than actively partaking in the creation of new art. Reading for pleasure has been previously reported as the most popular arts engagement activity in *Taking Part* (64% of the sample reported reading for pleasure [9]) and appears to increase overall rates of arts engagement when included as an item [6]. National surveys and epidemiological studies missing a question on listening to recorded music in particular, as well as explicit mentions of “podcasts” and “audiobooks”, ought therefore to consider inclusion in future data collection waves to avoid underrepresentation of arts engagement. Indeed, a recent report suggests steady increases in both free and paid music streaming services use (e.g. weekly engagement with music ranges between 42%-83% for Youtube and 6%-67% for Spotify depending on the age-group), as well as other digital audio content such as audiobooks and podcasts [15]. For instance, it has been estimated that approximately 12% (one in eight) of the UK population listens to podcasts on a weekly-basis [15], and this number matches closely to 10.92% weekly engagement with audiobooks and podcasts reported in our survey (a further 5.90% of respondents reported daily engagement).

Overall, we found high levels of arts engagement in our sample: 97.4% of all respondents reported engaging with at least one of the 20 arts activities once in the last year (2018–2019). On average, respondents reported engaging with 9.5 arts activties: 3.9 participatory arts and 5.6 receptive arts activties. Although we discourage direct comparisons with nationally-representative probability-based panel studies and national surveys, the rates found here are significantly higher than those found in *Understanding Society* (76% for participatory arts engagement and 61% for receptive arts engagement) and *Taking Part* (77.5% for overall arts engagement, 2013–2014 data). This higher engagement might be accounted for through our attempts to adapt the arts engagement scale to increase its contemporary relevance, inclusivity, and generalizability, as discussed above in relation to reading. However, higher rates of engagement found here are also likely to be due to a sampling bias inherent in most nonprobability-based online surveys. People who volunteer and engage with more community activities are also more likely to take part in surveys [78], and participation in surveys is influenced by the interest in the subject researched [79].

Quantifying arts engagement by deriving art engagement profiles, we found three distinct engagement clusters: *low engagers*, *receptive consumers*, and *omnivores*, comprising 19.8%, 44.4%, and 35.8% of the sample respectively. This study, in agreement with previous reports (e.g. [8]), demonstrates that people tend to mix different art forms in their overall arts engagement patterns. Our arts engagement cluster breakdown resembles that found in *Understand Society* 2010–2012 data for receptive arts and cultural engagement which also suggests three clusters: “rarely engaged”, “infrequently engaged”, and “frequently engaged” comprising 47.7%, 33.9%, and 18.4% of the UK population respectively [80]. Reports commissioned by Arts Council England and tested in 2006/07 and 2009/10 *Taking Part* data also identified three overall arts engagement segments: “not currently engaged”, “some engagement”, and “highly engaged” respectively comprising 23%, 68%, and 9% of the English population in 2006/2007 and 27%, 66%, and 7% in 2009/2010 [81, 82]. It is also of note that the patterns of engagement in the population are highly influenced by the definitions of engagement adopted [9]. For instance, using *Taking Part* data and broad definitions of cultural and social engagement, Taylor [9] identified that only 11% of the English population can be described as detached from mainstream cultural activities besides television watching. Taken together, both the summative score (only 2.57% not engaging with any of the 20 arts activities between 2018–2019) and cluster analysis (35.8% of the sample consisting of *omnivores*) show high levels of arts engagement in our sample. Both summative score and cluster analysis approaches to measuring arts engagement also confirm previously found differences in demographic, socioeconomic, and health characteristics, with higher engagement found in younger age groups (18–25 years of age in particular), women, those with higher education and higher income, better health, and in coupled relationships [6, 8] and, unlike previously reported, also in ethnic minorities.

To the best of our knowledge, this study is the first large-scale survey study to investigate the extent to which engagement with different art forms and activities occurs *mainly with others* or *mainly alone*, instead of relying on an untested assumptions of solitary or social activities [18, 19]. We found literary arts activities such as reading, writing, and listening to audiobooks and podcasts, as well as participatory visual arts such as painting or drawing and crafts, were engaged in *mainly alone*, while popular receptive arts activities such as live theater or circus, live music concerts, film screenings, exhibitions or museum, and live dance were engaged in *mainly with others*. The extent to which spending time alone or socializing with others is a primary purpose of engagement with these arts activities is unclear; however, the social setting appears to be an inevitable component of, in particular, certain receptive activities such as live theater or concerts. Indeed, our pooled results for participatory and receptive arts engagement further showed that overall engagement in receptive arts is mainly social (43.19%) or a mixture of social and solitary (32.10%), while engagement in participatory arts is mainly solitary (56.8%). Interestingly, the top two most popular and most frequently engaged in activities–reading and listening to recorded music–are predominantly solitary activities; however, *receptive consumers*, the largest art engagement segment in our sample, are predominantly comprised of social, receptive arts activities. The amount of time spent alone or with others as part of one’s arts engagement is likely to vary from individual to individual and may be important to understand when designing an arts-based intervention targeting social isolation and loneliness, especially for at-risk groups such as younger or older adults, which present different arts engagement profiles. Although future research is needed to explore this notion further, it may be possible that, for instance, a time-limited arts-based intervention targeting social isolation (e.g. museum-based programs for older adults [41]) may engender a more sustained benefit by choosing arts activities that are intrinsically social and therefore more conducive to continued social engagement outside the intervention compared with more traditionally solitary activities.

We further found that arts engagement correlated positively with both negative (depression) and positive (psychological wellbeing and social connectedness) aspects of mental and social wellbeing. The positive association between arts engagement and odds of depression was largely consistent across both art engagement measurement approaches. These findings are mostly in contradiction to previous cross-sectional studies of adult populations reporting no associations between depression and receptive and participatory arts engagement [83, 84] or a negative association [85], and similarly in longitudinal studies, no association between cultural (mainly receptive) events at work and symptoms of depression [86] or a negative association between receptive arts engagement and odds of depression in older adults [3]. Here, the overall summative arts engagement score was positively associated with odds of depression, and *omnivores* compared with *low engagers* had higher odds of depression, after adjusting for socioeconomic, health, and social covariates. The participatory arts engagement score was most strongly associated with higher odds of depression, while the positive association between depression and receptive arts engagement (whether measured through summative receptive arts engagement score or the comparison between *receptive consumers* and *omnivores*) was less robust due to apparent confounding by health and social covariates. One explanation for this finding may be that participatory arts activities were more likely to occur at a greater frequency and alone rather than with others. Affective reactions to solitude have been found to vary between individuals; however, evidence suggests that frequent solitude when coupled with high negative affect may contribute to risk of depression [87]. Taken together, these findings suggest that when examining the association between arts engagement and depression at the population-level, future studies ought to be aware that art engagement profiles have a distinct composition of specific participatory and receptive arts activities, which will differ in the intensity/frequency of engagement and in social context, and may confound the association.

It is also possible that the positive association between arts engagement and depression found in our sample represents people with pre-existing depressive symptoms using arts as a coping mechanism. In fact, previous studies suggest that arts engagement, such as music listening or drawing can be used for regulating depressed mood [88–90]. Furthermore, evidence in young adults suggests that reliance on music for emotional coping is more likely to occur during periods of low mood and that music listening or drawing when used as part of rumination or venting strategy may further increase depressive symptoms [88, 90, 91]. The evidence on the use of arts as a coping mechanism for depression is further strengthened by numerous examples of successful, mainly participatory arts-based interventions aimed at alleviating depressive symptoms in groups suffering from depression and other affective mental health disorders or preventing them in at risk groups, for instance singing workshops for mothers with postnatal depression [22] and older adults at risk of low mental health-related quality of life [92]. For a recent systematic review of participatory arts-based interventions targeting depression in older adults, see Dunphy et al. [93].

Moving to wellbeing, we found a consistent positive association between arts engagement and positive psychological wellbeing across the two measurement approaches, before and after covariate adjustment. This is largely in agreement with previous cross-sectional studies of population-based surveys reporting positive associations between arts engagement (in particular receptive arts engagement) and happiness, life satisfaction, and good quality of life [4, 85, 94–96], as well as the overall body of evidence on arts-based initiatives for wellbeing [12, 21]. The consistency across the two arts engagement measurement approaches showed no material differences in effect sizes between participatory and receptive arts engagement scores and larger effect sizes for *omnivores* compared with *receptive consumers*. This suggests that the association between arts engagement and wellbeing may be dose-response and that the wellbeing benefit of engagement may not be limited to a particular arts activity or activities. Given the cross-sectional nature of our study it is difficult to speculate on the direction of the association. Nonetheless, evidence from longitudinal and interventional studies predominantly including older adults provides some support for arts engagement contributing to increased wellbeing. For instance, arts-based intervention studies report short-term wellbeing benefits from participation in music making workshops, community group singing, and museum-based programs [92, 97, 98], while a longitudinal study using *ELSA* suggests that association between receptive arts engagement and hedonic and eudemonic wellbeing is most robust when the engagement is sustained over time [20] Lomas (2016) provides a review of processes through which the arts can support thriving [26].

In terms of social wellbeing, we found no association between arts engagement and loneliness measured through the most commonly used revised three-item short-form UCLA Loneliness scale [70, 99]. This scale has been repeatedly demonstrated to display satisfactory internal consistency, test-retest reliability, and concurrent and discriminant validity [70, 99–101], and its three-item short-form version (alongside a direct loneliness question) is currently the recommended loneliness measure at the national level in the UK [71]. However, this unidimensional global loneliness measure has also been criticized for its conceptual bias toward social dimensions of loneliness, such as inadequate social network, particularly experienced in separated groups such as “homesick” students and insufficient representation of emotional aspects of loneliness such as inadequate intimacy in relationships [100–102]. In some studies the UCLA scale has been outperformed by multidimensional instruments measuring most notably both social and emotional dimensions of loneliness such as the De Jong Gierveld Loneliness scale [100, 103, 104]. Therefore, we used the De Jong Gierveld scale in our sensitivity analyses, looking at global loneliness as well as emotional and social dimensions of loneliness. We found that *receptive consumers* and *omnivores* had lower odds of intense global loneliness compared with *low engagers*; however, this association was not replicated using summative arts engagement scores. It is possible that one’s overall arts engagement profile is a more accurate proxy of one’s social lifestyle which may be more closely linked with odds of feeling intensely lonely, than the number of arts activities or their receptive or participatory nature. The finding on global loneliness is broadly in agreement with a previous choir-based intervention study that reported a decrease in loneliness among the intervention group compared with a control group [105] and a longitudinal study using *ELSA* data reporting a dose-response negative association between receptive arts engagement at study baseline and odds of loneliness ten years later [14].

Across the two measurement approaches (overall, participatory, and receptive arts engagement scores vs. art engagement clusters), arts engagement was negatively associated with odds of intense social loneliness, with larger effect sizes for receptive arts engagement and *omnivores*. Taken together, these findings lead us to speculate that the frequency or intensity of engagement possibly through facilitating socializing may be more pertinent for lower likelihood of intense social loneliness than specific arts activities. The patterns of association for social loneliness were also largely reflected in the associations between arts engagement and social connectedness. Using summative scores, we found that receptive arts engagement was most robustly associated with social connectedness, and art cluster analysis showed that both *receptive consumers* and *omnivores* report higher social connectedness than *low engagers*, with the larger effect size for *omnivores* compared with *receptive consumers*. The findings on social loneliness and social connectedness are largely in agreement with the body of evidence from arts-based interventions (including singing, visual arts, and other creative arts workshops as well as museum-based programs) reporting increases in social interactions such as supportive exchanges, the number and closeness of social relationships, and the feeling of social inclusion and belonging [41, 44, 45, 97, 106–111]. Some of these interventions further suggest that singing is more effective at increasing perceived emotional closeness than talking [112] and that certain art activities such as singing and writing are most conducive to accelerating the pace of social bonding and feelings of connection in a group than others such as crafts [44].

Our finding on the positive association between all arts engagement summative scores and odds of intense emotional loneliness is unexpected and in contradiction to previous evidence. With a pattern that is similar to our findings on depression, participatory arts engagement was most robustly associated with higher odds of intense emotional loneliness, and *receptive consumers* seemed to have lower odds of intense emotional loneliness compared with *low engagers*. It may be possible that people who suffer from emotional loneliness use the arts, in particular more solitary participatory arts activities, as a means of emotional regulation and soothing, and that the feelings of social connectedness derived in particular from frequent, more social receptive arts activities may help to counteract feelings of intense emotional loneliness. Further research is needed to examine the differences in emotional and social dimensions of loneliness in relation to arts engagement.

Using this nonprobability online survey has enabled fast collection of new data currently missing in existing probability-based cohort studies. However, generalizability of our findings to the general population is limited. This online sample reported unusually low levels of non-engagement with the arts (2.57%) and high proportions of people reporting depression (57.04% scored ≥ 3 on CES-D scale) and loneliness (22.91% reporting feeling loneliness “often” or “always”), compared with approximately 20% of UK adults reporting anxiety or depression in 2014 [113] or 5% adults in England who reported feeling lonely “often” or “always” between 2016 and 2017 [34]. Inherent sampling bias of nonprobability online surveys attracting respondents interested in the subject and those with more prosocial and cooperative tendencies cannot be ruled out in our case [78, 79]. In addition, we encountered issues of non-response. Eleven percent of our respondents preferred “not to say” in response to at least one of the sensitive demographic questions. We followed Langkamp et al. [114] and excluded these cases. However, future work using the alternative methods for analysis of these data would clarify the extent of resulting bias. This is particularly important given the patterns of respondents who tend to provide demographic information in surveys less frequently [115]. Furthermore, our findings from linear regression models ought to be interpreted with caution due to significant departures from normal distribution and homoscedasticity of residuals for both wellbeing and social connectedness models, which we addressed by applying robust standard errors.

Several future research directions are suggested by this study in terms of population-level arts engagement assessment, its social context, and implications. Ongoing large-scale surveys may consider a more inclusive formulation of their existing arts engagement scales and update them with activities currently missing, in particular listening to recorded music as well as more explicit mentions of audiobooks and podcasts. Differences in terms of average engagement frequency patterns and social context between participatory and receptive arts activities suggests the importance of examining them separately and systematically in exploration of both arts engagement and its implications for psychological and social wellbeing. For instance, with the social distancing restrictions associated with the global pandemic of COVID-19, a future study is needed to explore whether arts engagement has shifted towards solitary, online engagement, and whether the solitary versus social nature of engagement division between participatory and receptive arts activities persists. Our results suggest that arts engagement may correlate positively with psychological wellbeing (including eudemonic and hedonic wellbeing) as well as subjective experiences of one’s social relationships such as perceived social connectedness; nonetheless, it can also be positively associated with depressive symptoms and feelings of emotional loneliness. In current global circumstances, the use of arts as a means of emotional loneliness soothing and coping with depressive symptoms warrants further investigation. Future research testing these associations in probability-based population-representative samples is needed.

Finally, this pattern of associations highlights the importance of considering the art form, type of engagement, frequency of engagement, social context, and demographic and wellbeing characteristics of participants in understanding how and why arts engagement may have positive effects in some cases and not others.

## Conclusion

This article has introduced the HEartS Survey, a tool for the exploration of arts engagement and the connection between these activities and social wellbeing. Based on data collected from 5,338 adults in the UK, we found that over 97% of respondents reported engagement in at least one arts activity at least once during 2018–2019, with reading and listening to music the most popular activities. Arts engagement grouped into three clusters: “low engagers” whose main source of engagement was occasional reading; “receptive consumers” who read and listened to music frequently and engaged with popular receptive arts activities, such as cinema, live music, theater, exhibitions, and museums; and “omnivores” who frequently engaged in almost all arts activities. In agreement with existing studies, more arts engagement was associated with higher levels of wellbeing, social connectedness, and lower odds of intense social loneliness. In contrast, we found a positive association between more arts engagement and depression and intense emotional loneliness for the most highly engaged omnivores. We conclude that arts engagement in the population forms specific profiles with distinct characteristics and that arts engagement is positively associated with better social wellbeing in most but not all cases. People who experience emotional loneliness may use the arts as a means of emotional regulation and soothing. Indeed, feelings of social connectedness derived in particular from frequent, more social receptive arts activities may help to counteract feelings of intense emotional loneliness.

## Supporting information

S1 Table Results from logistic regression analyses examining the association between arts engagement score and odds of loneliness using De Jong Gierveld loneliness scale. All outcomes were entered and analysed in individual models, UK-wide *HEartS Survey* n = 5,338.
(PDF)

S2 Table Results from logistic regression analyses examining the association between arts engagement clusters and odds of loneliness using De Jong Gierveld loneliness scale. All outcomes were entered and analysed in individual models, UK-wide *HEartS Survey*, n = 5,338.
(PDF)

S1 File *HEartS Survey*. Survey questions and titles of validated scales used.
(PDF)

S2 File Demographic questions in the survey and manipulations for analysis. (PDF)
