# Supplementary material for: Arts engagement trends in the United Kingdom and their mental and social wellbeing implications: HEartS Survey
Source: PLoS One. 2021 Mar 12;16(3):e0246078. doi: 10.1371/journal.pone.0246078 (PMC7954337; doi:10.1371/journal.pone.0246078)
Supplement: S1 Table — All outcomes were entered and analysed in individual models, UK-wide HEartS Survey n = 5,338. (PDF) [file pone.0246078.s001.pdf]

Tymoszuk U, Spiro N, Perkins R, Mason-Bertrand A, Gee K, and Williamon A (2021), Arts Engagement Trends in the UK and Their Mental and Social Wellbeing Implications: HEartS Survey, *PLOS One* 16:e0246078, doi: 10.1371/journal.pone.0246078.

S1 TABLE | Results from logistic regression analyses examining the association between arts engagement score and odds of loneliness using De Jong Gierveld Loneliness Scale. All arts engagement variables and all outcomes were entered and analysed in individual models, UK-wide *HEartS Survey*, n=5,338.

	**De Jong Gierveld Loneliness Scale**											
	**Global loneliness**				**Emotional loneliness**				**Social loneliness**			
	**OR**	**95% CI**	***p***	**R^2**	**OR**	**95% CI**	***p***	**R^2**	**OR**	**95% CI**	***p***	**R^2**
Model 1: sociodemographic variables												
Overall arts activities score	0.98	0.97–1.00	*0.015*	3.10%	1.03	1.02–1.04	*<0.001*	6.62%	0.95	0.94–0.96	*<0.001*	1.98%
Participatory arts activities score	0.99	0.97–1.01	*0.44*	3.00%	1.07	1.05–1.09	*<0.001*	6.89%	0.94	0.92–0.96	*<0.001*	1.31%
Receptive arts activities score	0.96	0.94–0.98	*<0.001*	3.24%	1.03	1.01–1.04	*0.014*	6.36%	0.91	0.89–0.92	*<0.001*	2.33%
Model 2: Model 1 + socioeconomic variables												
Overall arts activities score	0.99	0.98–1.00	*0.18*	4.42%	1.04	1.02–1.05	*<0.001*	8.22%	0.95	0.94–0.96	*<0.001*	2.34%
Participatory arts activities score	1.00	0.97–1.02	*0.83*	4.39%	1.08	1.06–1.10	*<0.001*	8.41%	0.94	0.92–0.96	*<0.001*	1.74%
Receptive arts activities score	0.98	0.95–1.00	*0.031*	4.48%	1.04	1.02–1.07	*<0.001*	7.93%	0.91	0.89–0.93	*<0.001*	2.61%
Model 3: Model 2 + health and fitness variables												
Overall arts activities score	0.99	0.98–1.01	*0.37*	8.77%	1.04	1.02–1.05	*<0.001*	12.27%	0.96	0.95–0.97	*<0.001*	5.23%
Participatory arts activities score	1.00	0.97–1.02	*0.85*	8.75%	1.08	1.05–1.10	*<0.001*	12.37%	0.95	0.93–0.97	*<0.001*	4.87%
Receptive arts activities score	0.98	0.96–1.01	*0.16*	8.79%	1.05	1.02–1.07	*<0.001*	12.04%	0.92	0.91–0.94	*<0.001*	5.40%
Model 4: Model 3 + social circumstances variables												
Overall arts activities score	0.99	0.98–1.01	*0.24*	11.30%	1.04	1.02–1.05	*<0.001*	15.07%	0.96	0.94–0.97	*<0.001*	8.26%
Participatory arts activities score	0.99	0.96–1.02	*0.50*	11.28%	1.07	1.05–1.10	*<0.001*	15.13%	0.94	0.92–0.96	*<0.001*	7.88%
Receptive arts activities score	0.98	0.96–1.01	*0.15*	11.31%	1.05	1.02–1.07	*<0.001*	14.88%	0.92	0.90–0.94	*<0.001*	8.37%

Abbreviations: OR, odds ratio; CI, confidence interval; R^2, Pseudo R-squared in logistic regression models and R-squared in linear regression models

Cut points for cases: intense global loneliness = max score of 6; intense emotional loneliness = max score of 3; intense social loneliness = max score of 3

Model 1: gender, age, ethnicity; *Model 2:* Model 1 + educational attainment, household income; *Model 3:* Model 2 + self-rated health, physical activity (mild, moderate, vigorous); *Model 4:* Model 3 + living status, closeness of relationship with a partner
