# Supplementary material for: Arts engagement trends in the United Kingdom and their mental and social wellbeing implications: HEartS Survey
Source: PLoS One. 2021 Mar 12;16(3):e0246078. doi: 10.1371/journal.pone.0246078 (PMC7954337; doi:10.1371/journal.pone.0246078)
Supplement: S2 Table — All outcomes were entered and analysed in individual models, UK-wide HEartS Survey, n = 5,338. (PDF) [file pone.0246078.s002.pdf]

Tymoszuk U, Spiro N, Perkins R, Mason-Bertrand A, Gee K, and Williamon A (2021), Arts Engagement Trends in the UK and Their Mental and Social Wellbeing Implications: HEartS Survey, *PLOS One* 16:e0246078, doi: 10.1371/journal.pone.0246078.

S2 TABLE | Results from logistic regression analyses examining the association between arts engagement clusters and odds of loneliness using De Jong Gierveld Loneliness Scale. All outcomes were entered and analysed in individual models, UK-wide *HEartS Survey*, n=5,338.

	**De Jong Gierveld Loneliness Scale**											
	**Global loneliness**				**Emotional loneliness**				**Social loneliness**			
	**OR**	**95% CI**	***p***	**R^2**	**OR**	**95% CI**	**p**	**R^2**	**OR**	**95% CI**	***p***	**R^2**
Model 1: sociodemographic variables (reference: low engagers)												
Receptive consumers	0.70	0.59–0.84	*<0.001*	3.39%	0.68	0.58–0.81	*<0.001*	6.64%	0.74	0.64–0.85	*<0.001*	1.64%
Omnivores	0.65	0.54–0.79	*<0.001*		0.92	0.77–1.09	*0.31*		0.53	0.45–0.62	*<0.001*	
Model 2: Model 1 + socioeconomic variables (reference: low engagers)												
Receptive consumers	0.75	0.62–0.89	*0.001*	4.62%	0.74	0.62–0.87	*<0.001*	8.01%	0.75	0.65–0.87	*<0.001*	2.01%
Omnivores	0.72	0.60–0.88	*0.001*		1.02	0.86–1.21	*0.84*		0.54	0.46–0.64	*<0.001*	
Model 3: Model 2 + health and fitness variables (reference: low engagers)												
Receptive consumers	0.79	0.66–0.96	*0.016*	8.90%	0.78	0.65–0.93	*0.007*	12.01%	0.82	0.70–0.96	*0.011*	5.06%
Omnivores	0.75	0.61–0.93	*0.007*		1.03	0.85–1.24	*0.79*		0.61	0.51–0.72	*<0.001*	
Model 4: Model 3 + social circumstances variables (reference: low engagers)												
Receptive consumers	0.80	0.66–0.97	*0.021*	11.41%	0.79	0.66–0.95	*0.011*	14.86%	0.85	0.71–0.98	*0.024*	7.96%
Omnivores	0.76	0.61–0.94	*0.010*		1.04	0.86–1.26	*0.68*		0.61	0.51–0.73	*<0.001*	

Abbreviations: OR, odds ratio; CI, confidence interval; R^2, Pseudo R-squared

Cut points for cases: intense global loneliness = max score of 6; intense emotional loneliness = max score of 3; intense social loneliness = max score of 3

Model 1: gender, age, ethnicity; *Model 2:* Model 1 + educational attainment, household income; *Model 3:* Model 2 + self-rated health, physical activity (mild, moderate, vigorous); *Model 4:* Model 3 + living status, closeness of relationship with a partner
