# Supplementary material for: Arts engagement trends in the United Kingdom and their mental and social wellbeing implications: HEartS Survey
Source: PLoS One. 2021 Mar 12;16(3):e0246078. doi: 10.1371/journal.pone.0246078 (PMC7954337; doi:10.1371/journal.pone.0246078)
Supplement: S1 File — Survey questions and titles of validated scales used. (PDF) [file pone.0246078.s003.pdf]

HEartS
Health, Economic, and Social impact of the ARTs

1. Welcome and consent
2. Demographic questions
3. Arts participation
4. Arts reception
5. Other arts engagement
6. Open response
7. Wellbeing and physical activity
8. Social connection and loneliness
9. Household income and arts spending
10. Further information

HEartS Survey 2019

Note

Responses were collected through the Qualtrics online platform over a period of six months: March–August 2019.
Previously published scales are named here but not reproduced in full.

Dataset

Williamon A, Tymoszuk U, Spiro N, Gee K, Mason-Bertrand A, and Perkins R (2021), HEartS Survey 2019: Charting the Health, Economic, and Social impact of the ARTs, *Dryad*, Dataset, doi: 10.5061/dryad.3r2280gdj.

Report

Tymoszuk U, Spiro N, Perkins R, Mason-Bertrand A, Gee K, and Williamon A (2021), Arts Engagement Trends in the UK and Their Mental and Social Wellbeing Implications: HEartS Survey, *PLOS One* 16:e0246078, doi: 10.1371/journal.pone.0246078.

Correspondence

Aaron Williamon, aaron.williamon@rcm.ac.uk

Funded by the Arts and Humanities Research Council (AHRC) | United Kingdom
Grant Ref. AH/P005888/1

1. Welcome and consent

Welcome to the survey

Thank you for considering taking part in our research. We are interested in your creative, artistic, cultural pursuits, and your wellbeing. For now, this survey is limited to people who currently live in the UK and are 18 or over. Please only take part in this survey once. Please note, not all of the pages contain a back button.

Consent

Your participation in this research is voluntary, and you may withdraw from the study at any time if you wish. By clicking 'next' below, however, you are giving your informed consent to participate in the study.

What will we do with your data?

The data you provide will be anonymous (separated from your name) and confidential (not disclosed to anyone else). The data will be stored securely at Imperial College London and the Royal College of Music according to the Colleges' Data Retention Policies, and used as part of the HEartS project which is funded by the Arts and Humanities Research Council to explore the health and social impact of the arts. We plan to publish portions of our data set, reports, articles and presentations based on our findings, but you will not be identifiable from the data.

Who should I contact if I have queries about the research?

If you have any queries or concerns about the research, you can contact our research team at hearts@rcm.ac.uk. The project has been reviewed and approved by the Research Ethics Committee of Conservatoires UK.

We will be asking you questions about your mental health and wellbeing. Therefore, at the end of the questionnaire, there will be links to sources of information and support on these issues.

1.1 By checking the three boxes below you are giving your informed consent to participate in the study and confirming that:

- ❑ This is the first time you are completing this survey
- ❑ You are living in the UK
- ❑ You are 18 years or older

2. Demographic questions

2.1 In which region do you live?

- ❑ Northern Scotland
- ❑ Southern Scotland
- ❑ North East
- ❑ North West
- ❑ Yorkshire and the Humber
- ❑ East Midlands
- ❑ West Midlands
- ❑ East of England
- ❑ South East
- ❑ South West
- ❑ London
- ❑ North Wales
- ❑ South Wales
- ❑ Northern Ireland

2.2 What is your ethnic group?

- ❑ White - English / Welsh / Scottish / Northern Irish / British
- ❑ White - Irish
- ❑ White - Gypsy or Irish Traveller
- ❑ Any other White Background
- ❑ Mixed / Multiple ethnic groups - White and Black Caribbean
- ❑ Mixed / Multiple ethnic groups - White and Black African
- ❑ Mixed / Multiple ethnic groups - White and Asian
- ❑ Any other Mixed / Multiple ethnic background
- ❑ Asian / Asian British - Indian
- ❑ Asian / Asian British - Pakistani
- ❑ Asian / Asian British - Bangladeshi
- ❑ Asian / Asian British - Chinese
- ❑ Any other Asian background
- ❑ Black / African / Caribbean / Black British - African
- ❑ Black / African / Caribbean / Black British - Caribbean
- ❑ Any other Black / African / Caribbean background
- ❑ Arab
- ❑ Any other ethnic group

2.3 What is the highest educational or school qualification you have obtained?

- ❑ University higher degree (e.g. masters or doctoral studies, MA, MSc, PhD)
- ❑ First degree level qualification (e.g. Bachelor's degrees, foundation degrees, graduate membership of a professional institute, PGCE, diploma in Higher Education, nursing or other medical qualification)
- ❑ National Vocational Qualification (NVQ) / Scottish Vocational Qualifications (SVQ) - Level 4-5
- ❑ A and AS Level, Welsh or international baccalaureate, or equivalent
- ❑ National Vocational Qualification (NVQ) / Scottish Vocational Qualifications (SVQ) - Level 3
- ❑ GCSE, O Level, AS Level, CSE, Higher grade or standard ordinary
- ❑ National Vocational Qualification (NVQ) / Scottish Vocational Qualifications (SVQ) - Level 1-2
- ❑ Other vocational qualification (e.g. apprenticeship, youth training certification, HNC/HND, ONC/OND, BTEC, SCOTVEC, City and Guilds)
- ❑ Other qualifications including foreign qualifications
- ❑ No formal qualifications
- ❑ Would rather not say

2.4 I identify myself as

- ❑ Male
- ❑ Female
- ❑ Would rather not say
- ❑ Other (please define) ____________________________________

2.5 How old are you?

(Please write in numbers, e.g. 42) ________

2.6 With whom do you live? (tick all that apply)

- ❑ On my own
- ❑ Residential care setting
- ❑ My spouse or partner
- ❑ Children
- ❑ Other family
- ❑ Friends or house share
- ❑ Other
- ❑ Would rather not say

2.7 Do you work as a professional artist, performer, or maker in any of the following arts and cultural areas?

- ❑ Literature (e.g. professional writer, publisher, poet etc.)
- ❑ Music or sound arts (e.g. professional musician)
- ❑ Visual arts (e.g. professional photographer, graphic designer, artist etc.)
- ❑ Crafts (e.g. professional who works with textiles, wood or metal crafts, pottery or ceramics, or jewellery making)
- ❑ Decorative arts (e.g. professional florist, gardener, chef / cook)
- ❑ Performing arts (e.g. actor, dancer, circus performer)
- ❑ No, I do not work professionally in the arts

3. Arts participation

This section explores the types of arts and cultural activities that you do **as a participant** as a past-time activity, i.e. where you are actively involved in doing, creating, making, etc. rather than as a spectator, listener, or audience member.

3.1 In the last 12 months how often have you done any of the following activities?

	Never	A one off engagement	Once or twice a year	Every few months	Monthly	Weekly	Daily
Read as a past-time activity	❑	❑	❑	❑	❑	❑	❑
Written as a past-time activity	❑	❑	❑	❑	❑	❑	❑
Attended a book club where people meet to discuss and share books or stories	❑	❑	❑	❑	❑	❑	❑
Played a musical instrument or sang	❑	❑	❑	❑	❑	❑	❑
Written or created music	❑	❑	❑	❑	❑	❑	❑
Practised, rehearsed or performed dance	❑	❑	❑	❑	❑	❑	❑
Practised, rehearsed or performed a play, drama, opera, operetta, musical theatre, circus skills	❑	❑	❑	❑	❑	❑	❑
Done photography, film, video making, or other related pursuits	❑	❑	❑	❑	❑	❑	❑
Done painting, drawing, printmaking, sculpture, or other related pursuits	❑	❑	❑	❑	❑	❑	❑
Done any form of crafts including textile crafts, wood or metal crafts, pottery or ceramic crafts, jewellery or any other decorative arts	❑	❑	❑	❑	❑	❑	❑

3.2 Do you usually undertake these activities alone or with other people?

{Logic based on 3.1}	Mainly alone	Alone and with others	Mainly with others
Read as a past-time activity	❑	❑	❑
Written as a past-time activity	❑	❑	❑
Attended a book club where people meet to discuss and share books or stories	❑	❑	❑
Played a musical instrument or sang	❑	❑	❑
Written or created music	❑	❑	❑
Practised, rehearsed or performed dance	❑	❑	❑
Practised, rehearsed or performed a play, drama, opera, operetta, musical theatre, circus skills	❑	❑	❑
Done photography, film, video making, or other related pursuits	❑	❑	❑
Done painting, drawing, printmaking, sculpture, or other related pursuits	❑	❑	❑
Done any form of crafts including textile crafts, wood or metal crafts, pottery or ceramic crafts, jewellery or any other decorative arts	❑	❑	❑

4. Arts reception

This section explores the types of past-time arts and cultural activities that you do **as a spectator, listener, or audience member**, rather than as a participant.

4.1 In the last 12 months how often have you done any of the following activities?

	Never	A one off engagement	Once or twice a year	Every few months	Monthly	Weekly	Daily
Been to an event connected with books or reading	❑	❑	❑	❑	❑	❑	❑
Listened to audio books or podcasts	❑	❑	❑	❑	❑	❑	❑
Been to live music	❑	❑	❑	❑	❑	❑	❑
Listened to recorded music (not including music you happen to overhear)	❑	❑	❑	❑	❑	❑	❑
Been to live dance	❑	❑	❑	❑	❑	❑	❑
Been to live theatre or circus	❑	❑	❑	❑	❑	❑	❑
Watched a film or drama at a cinema or other venue outside of the home	❑	❑	❑	❑	❑	❑	❑
Been to an exhibition, museum, or collection of art, photography, sculpture or any other arts	❑	❑	❑	❑	❑	❑	❑
Been to a convention, show, or fair relating to crafts or decorative arts	❑	❑	❑	❑	❑	❑	❑
Been to street art, public art displays, visited archi-tecture or an historical monument (not including art or architecture that you happen to see)	❑	❑	❑	❑	❑	❑	❑

4.2 Do you usually undertake these activities alone or with other people?

{Logic based on 4.1}	Mainly alone	Alone and with others	Mainly with others
Been to an event connected with books or reading	❑	❑	❑
Listened to audio books or podcasts	❑	❑	❑
Been to live music	❑	❑	❑
Listened to recorded music (not including music you happen to overhear)	❑	❑	❑
Been to live dance	❑	❑	❑
Been to live theatre or circus	❑	❑	❑
Watched a film or drama at a cinema or other venue outside of the home	❑	❑	❑
Been to an exhibition, museum, or collection of art, photography, sculpture or any other arts	❑	❑	❑
Been to a convention, show, or fair relating to crafts or decorative arts	❑	❑	❑
Been to street art, public art displays, visited archi-tecture or an historical monument (not including art or architecture that you happen to see)	❑	❑	❑

5. Other arts engagement

5.1 Have you engaged in any other participatory or receptive arts or cultural activities not listed previously in this survey?

- ❑ Yes
- ❑ No

If yes

5.2 Please tell us the name of the arts or cultural activity which was not listed_________________________

5.3 How often have you engaged in this activity?

- ❑ A one off engagement
- ❑ Once or twice a year
- ❑ Every few months
- ❑ Monthly
- ❑ Weekly
- ❑ Daily

5.4 Do you usually undertake this activity alone or with other people?

- ❑ Mainly alone
- ❑ Alone and with others
- ❑ Mainly with others

6. Open response

6.1 Does your engagement with the arts and cultural activities you have told us about today help you to feel connected with other people?

{Logic based on 3.1, 4.1, and 5.1}

- ❑ Not at all
- ❑ A little
- ❑ Around half of the time
- ❑ Often
- ❑ Always

If a little, around half of the time, often, or always

> 6.2 Of the arts and cultural activities you have told us about, which makes you feel most connected to other people? (Please select just one activity)
>
> {Logic based on 3.1, 4.1, and 5.2}

6.3 Why does this activity make you feel connected to other people? Please write in as much detail as possible and include examples or stories where appropriate.

____

____

____

____

7. Wellbeing and physical activity

We now have some questions we would like you to answer on your wellbeing.

7.1 The Mental Health Continuum Short Form 14-item scale

7.2 Centre for Epidemiologic Study Depression scale (CES-D) short form 8-item scale

7.3 How is your health in general?

- ❑ Very good
- ❑ Good
- ❑ Fair
- ❑ Bad
- ❑ Very bad
- ❑ Would rather not say

7.4 Do you consider yourself as someone living with... (please tick all that apply)

- ❑ Mental health issues
- ❑ Cancer
- ❑ Cardiovascular disease
- ❑ Chronic respiratory disease
- ❑ None of the above
- ❑ Would rather not say

7.5 Physical activity scale in the Whitehall II Study

7.6 Do you usually take part in these **sports or other energetic activities** alone or with other people?

- ❑ Mainly alone
- ❑ Alone and with others
- ❑ Mainly with others

8. Social connection and loneliness

You are almost at the end of the survey! Please answer a few questions on your connections to other people.

8.1 Social Connectedness Revised 15-item scale

8.2 How many of your friends would you say you have a close relationship with?

- ❑ None
- ❑ One
- ❑ Two
- ❑ Three to four
- ❑ Five to eight
- ❑ Nine or more

8.3 How many of your family members would you say you have a close relationship with?

- ❑ None
- ❑ One
- ❑ Two
- ❑ Three to four
- ❑ Five to eight
- ❑ Nine or more

8.4 How close is your relationship with your partner or spouse?

- ❑ Not applicable. I don't have a partner or spouse.
- ❑ Not at all close
- ❑ Not very close
- ❑ Quite close
- ❑ Very close

8.5 UCLA Loneliness Short Form 4-item scale

8.6 De Jong Gierveld Loneliness Short Form 6-item scale

8.7 How often do you feel lonely?

- ❑ Always
- ❑ Often
- ❑ Sometimes
- ❑ Occasionally
- ❑ Hardly ever
- ❑ Never

8.8 How intense is this feeling?

- ❑ Not intense at all
- ❑ A little intense
- ❑ Neutral
- ❑ Quite intense
- ❑ Very intense

8.9 Inclusion of Other in Self Scale

9. Household income and arts spending

9.1 What is your household's income from all sources over the last 12 months?

Count income from every person included in the household. Include:

All earnings (include overtime, tips, bonuses, self-employment)
All pensions (include all student grants and bursaries but not loans)
All benefits and tax credits (such as child benefit, income support or pension credit)
All interest from savings or investments
All rent from property (after expenses)
Other income (such as maintenance or grants)

Do not deduct taxes, National Insurance contributions, Health Insurance payments, Superannuation payments.

❑ Up to £5,199
❑ £5,200 and up to £10,399
❑ £10,400 and up to £15,599
❑ £15,600 and up to £20,799
❑ £20,800 and up to £25,999
❑ £26,000 and up to £31,199
❑ £31,200 and up to £36,399
❑ £36,400 and up to £41,599
❑ £41,600 and up to £46,799
❑ £46,800 and up to £51,999
❑ £52,000 and up to £75,999
❑ £76,000 and above
❑ Would rather not say

9.2 In the **last month**, approximately how much has been spent on your arts and cultural activities?

This could include: online streaming subscriptions and services downloads, cinema, theatre, galleries, creative or artistic hobbies or events.

	Approximate monthly spend (£)<br>0 50 100 150 200
Arts and cultural activities	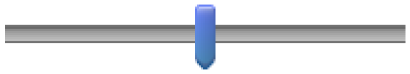

9.3 Reflecting on how much you have spent on arts and cultural activities in **the last year**, do you expect this to change in the **next 12 months**?

❑ I expect spending to **stay the same**
❑ I expect spending to **increase**
❑ I expect spending to **decrease**

If increase or decrease

9.4 You selected an increase or decrease in spending, please briefly explain why this might be the case.

____

____

____

____

10. Further information

Thank you for your help! In 15 seconds you will automatically be redirected to the final page to process your payment.

If this survey has raised any issues for you, please right hand click any of the hyperlinks below (to open in a new tab) which will take you to various support sites.

Sources of support:

It is important to seek help from your GP if you feel unhappy, sad or anxious, or if the thought of harming yourself or others has occurred to you. If you think there's a danger of imminent harm to you or others call or visit your local A&E services and ask to speak to the duty psychiatrist.

You can find information and support regarding wellbeing and mental health at the following links: NHS - Five steps to mental wellbeing; How to deal with stress; NHS - Stress and anxiety companion; Samaritans; Mind.

If you have any questions regarding this survey please contact our research team at hearts@rcm.ac.uk. More information about the study can be found here.
