# Supplementary material for: Arts engagement trends in the United Kingdom and their mental and social wellbeing implications: HEartS Survey
Source: PLoS One. 2021 Mar 12;16(3):e0246078. doi: 10.1371/journal.pone.0246078 (PMC7954337; doi:10.1371/journal.pone.0246078)
Supplement: S2 File — (PDF) [file pone.0246078.s004.pdf]

S2 File

Tymoszuk U, Spiro N, Perkins R, Mason-Bertrand A, Gee K, and Williamon A (2021), Arts Engagement Trends in the UK and Their Mental and Social Wellbeing Implications: HEartS Survey, *PLOS One* 16:e0246078, doi: 10.1371/journal.pone.0246078.

SUPPORTING INFORMATION S2 | Demographic questions in the *HEartS Survey* and manipulations for analysis.

Region (see supporting information S1, *HEartS Survey*, question 2.1)

UK geographic regions included: Northern Scotland; Southern Scotland; North East; North West; Yorkshire and the Humber; East Midlands; West Midlands; East of England; South East; South West; London; North Wales; South Wales; Northern Ireland.

Data were collapsed into four countries (England, Northern Ireland, Scotland, Wales) for analysis.

Ethnicity (see supporting information S1, HEartS Survey, question 2.2)

The list of ethnicities included: White – English / Welsh / Scottish / Northern Irish / British; White – Irish; White – Gypsy or Irish Traveller; Any other White Background; Mixed / Multiple ethnic groups – White and Black Caribbean; Mixed / Multiple ethnic groups – White and Black African; Mixed / Multiple ethnic groups – White and Asian; Any other Mixed / Multiple ethnic background; Asian / Asian British – Indian; Asian / Asian British – Pakistani; Asian / Asian British – Bangladeshi; Asian / Asian British – Chinese; Any other Asian background; Black / African / Caribbean / Black British – African; Black / African / Caribbean / Black British – Caribbean; Any other Black / African / Caribbean background; Arab; Any other ethnic group.

Due to small numbers in Black, Asian, and other ethnic minority groups, these data were combined for analysis.

Educational qualifications (see supporting information S1, HEartS Survey, question 2.3)

Education qualifications, and groupings for analysis, included:

- University higher degree (e.g. masters or doctoral studies, MA, MSc, PhD); First degree level qualification (e.g. Bachelor's degrees, foundation degrees, graduate membership of a professional institute, PGCE, diploma in Higher Education, nursing or other medical qualification); National Vocational Qualification (NVQ) / Scottish Vocational Qualifications (SVQ) – Level 4-5
- A and AS Level, Welsh or international baccalaureate, or equivalent; National Vocational Qualification (NVQ) / Scottish Vocational Qualifications (SVQ) – Level 3
- GCSE, O Level, AS Level, CSE, Higher grade or standard ordinary; National Vocational Qualification (NVQ) / Scottish Vocational Qualifications (SVQ) – Level 1-2
- Other vocational qualification (e.g. apprenticeship, youth training certification, HNC/HND, ONC/OND, BTEC, SCOTVEC, City and Guilds); Other qualifications including foreign qualifications; No formal qualifications

Gender (see supporting information S1, HEartS Survey, question 2.4)

In analyses, gender is used as a binary variable (male/female) due to small numbers in other gender categories; however, the survey allowed two further options, 'would rather not say' and 'other (please define)', to ensure inclusivity.

Age (see supporting information S1, HEartS Survey, §2.5)

Current age, as a number, was self-reported.

Living situation (see supporting information S1, HEartS Survey, question 2.6)

Living situation included eight multiple choice options which were collapsed into: living alone; living with partner; living with partner and children; living with family, house share, and other.

Professional work in the arts (see supporting information S1, HEartS Survey, question 2.7)

The demographic section closed by asking the respondent whether they worked professionally in the arts or cultural sphere including: literature (e.g. professional writer, publisher, poet etc.); music or sound arts (e.g. professional musician); visual arts (e.g. professional photographer, graphic designer, artist etc.); crafts (e.g. professional who works with textiles, wood or metal crafts, pottery or ceramics, or jewellery making); decorative arts (e.g. professional florist, gardener, chef / cook); performing arts (e.g. actor, dancer, circus performer).
